# Long noncoding RNAs in lipid metabolism: literature review and conservation analysis across species

**DOI:** 10.1186/s12864-019-6093-3

**Published:** 2019-11-21

**Authors:** Kevin Muret, Colette Désert, Laetitia Lagoutte, Morgane Boutin, Florence Gondret, Tatiana Zerjal, Sandrine Lagarrigue

**Affiliations:** 1 PEGASE, INRA, AGROCAMPUS OUEST, 35590 Saint-Gilles, France; 2 GABI INRA, AgroParisTech, Université Paris-Saclay, Domaine de Vilvert, 78352 Jouy-en-Josas, France

**Keywords:** lncRNA, Lipid metabolism, Liver, Evolution, Synteny

## Abstract

**Background:**

Lipids are important for the cell and organism life since they are major components of membranes, energy reserves and are also signal molecules. The main organs for the energy synthesis and storage are the liver and adipose tissue, both in humans and in more distant species such as chicken. Long noncoding RNAs (lncRNAs) are known to be involved in many biological processes including lipid metabolism.

**Results:**

In this context, this paper provides the most exhaustive list of lncRNAs involved in lipid metabolism with 60 genes identified after an in-depth analysis of the bibliography, while all “review” type articles list a total of 27 genes. These 60 lncRNAs are mainly described in human or mice and only a few of them have a precise described mode-of-action. Because these genes are still named in a non-standard way making such a study tedious, we propose a standard name for this list according to the rules dictated by the HUGO consortium. Moreover, we identified about 10% of lncRNAs which are conserved between mammals and chicken and 2% between mammals and fishes. Finally, we demonstrated that two lncRNA were wrongly considered as lncRNAs in the literature since they are 3′ extensions of the closest coding gene.

**Conclusions:**

Such a lncRNAs catalogue can participate to the understanding of the lipid metabolism regulators; it can be useful to better understand the genetic regulation of some human diseases (obesity, hepatic steatosis) or traits of economic interest in livestock species (meat quality, carcass composition). We have no doubt that this first set will be rapidly enriched in coming years.

## Background

Lipids are found in all organisms and are essential for life [1–3]. They contribute to cell membrane constitution, acting as signaling molecules and as an important source of energy. In animal species, the deregulation of lipid homeostasis processes is responsible for dyslipidemia and many diseases of major importance in human health such as obesity, diabetes, non-alcoholic fatty liver disease (NAFLD) or cardiovascular diseases. The major site of lipid synthesis may differ from one species to another. Lipogenesis from carbohydrates sources occurs mainly in the liver in primates and rodents, and in adipose tissue in carnivores and ungulates (pig, cow, goat, dog) [1]. Note that for chicken [4–6] and many fishes [7], the major site of de novo fatty acid synthesis is the liver. The synthesis of cholesterol in mammals, birds and fish is more ubiquitous but predominates in the liver [5, 8–10]. The storage of fatty acids in triglycerides is the main animal’s energy reserve, which is constituted during periods of energy excess and mobilized during periods of energy deprivation. This storage mainly occurs in adipose tissue within the adipocytes. Energy homeostasis is regulated by hypothalamus which regulates food intake and energy expenditure [11], and involves many hormones and adipokines to organize the crosstalk between organs. Lipid metabolism is a complex process that involves a large variety of molecular pathways and different transcriptional regulators some of which have been described so far.

Since the 2000s, with the advent of whole-genome sequencing technologies, most genes coding essentially for proteins have gradually been identified. The databases reference nearly 20,000 genes in human [12] [Human GENCODE v.28–10 July 2018], 22,000 in mouse [13] [Mouse GENCODE v.M17–10 July 2018] and pig [Ensembl v.92–10 July 2018], 18,000 in chicken [Ensembl v.92–10 July 2018]. Small RNAs, such as miRNA that do not code for proteins, are also relatively well annotated today (from 1705 to 5531). In contrast, long noncoding RNA (lncRNA) are less described in the genomes: they are strictly defined as RNAs of more than 200 nt with no ORF long enough to be translated into a protein (< 150 nt) [14]. They have important roles since they control the regulation of gene expression via a large diversity of mechanisms as they can interact with DNA, RNA or proteins [15, 16]. The number of lncRNAs with a well-described functional role is now estimated at 1% [17]. lncRNA genes appear to be as numerous as or even more numerous than genes encoding proteins with 15,779 and 12,533 lncRNA referenced via the GENCODE projects [12–14] in human [Human GENCODE v.28] and mouse [Mouse GENCODE v.M17], respectively. Modeling of these gene entities structure is far from complete. In human, specialized databases such as NONCODE [18] or LNCipedia [19] announce even higher numbers than those referenced in Ensembl and NCBI databases. On the contrary, in less studied livestock species such as pig and chicken [Ensembl v.92], a very small fraction of lncRNA is referenced (361 and 4643, respectively). So far, most lncRNA and their underlying transcripts, are gene models predicted by bioinformatics pipelines from RNAseq data and in the majority of species have rarely been experimentally confirmed. The difficulty of lncRNA gene modeling and identification is due to different causes: 1. an expression 10 to 100 times weaker than the protein coding genes [14, 20, 21], which makes harder the identification of the different transcripts exon-intron structures and therefore of the gene locus; 2. the tissue-specificity of lncRNA expression [14, 20], requiring the use of many different tissues to establish an exhaustive catalogue; 3. the criteria used for lncRNA prediction that can be the presence of an ORF, a blast against a protein database, the size of transcripts and/or the composition in *k-mers*, depending on the bioinformatics tool used; the main tools being CPC [22, 23], CPAT [24], PhyloCSF [25], FEELnc [26] and the Ulitsky’s team pipeline PLAR [27]. 4. Finally, a last point concerns the low conservation of lncRNA sequences between species, especially for species that are evolutionary distant [14, 20, 27–30]. All these drawbacks make it difficult to find orthologous long noncoding genes by sequence conservation analysis, as frequently done for protein-coding genes.

The first review about lncRNAs involved in lipid metabolism is very recent [31] (2016). Since then several other reviews and articles were published but none of them provided an exhaustive lipid-related lncRNA catalogue. The first objective of this study was to fill this gap providing an exhaustive catalogue of lncRNAs involved in lipid metabolism. An extensive analysis of the literature, generally focusing on human or mouse, allowed us to draw up a catalogue of 60 lncRNA genes related to lipid metabolism for which we report mechanisms of action when it is described This led us to highlight some spurious genes and therefore, to rename some lncRNAs in accordance to the rules published by the HUGO Gene Nomenclature Committee for long noncoding genes [32]. Second, we analyzed the conservation of these 60 lncRNA in chicken, which last shared common ancestor with mammal dates back to 300 M years ago, with the assumption that such a conservation would support an important role of these genes in the metabolism of interest. For this, an approach by synteny analysis was used, which highlighted 5 lncRNAs preserved between human/mouse and chicken. Finally, we have more precisely described their functional roles and analyzed their conservation between 8 species from mammals to zebrafish.

## Results

### 60 lncRNA identified as involved in lipid metabolism by expert curation of the literature

To our knowledge, the first catalogue of lncRNA potentially involved in lipid metabolism was proposed by Chen in 2016 [31] and included the 5 lncRNA, called *lncLSTR*, *HULC*, *APOA1-AS*, *lincRNA-DYNLRB2–2* and *SRA*. The same year, Zhou et al. [33] published a broader review of lncRNA genes potentially involved in lipid and glucose metabolisms and related diseases (atherosclerosis, type 2 diabetes, insulin resistance) in which *TRIBAL*, *ANRIL*, *lncLSTR*, *AT102202*, *APOA1-AS*, *lincRNA-DYNLRB2–2*, *RP5-833A20.1* and *CRNDE* were described as involved in lipid metabolism. Later in the year, Smekalova et al. [34] published a list of lncRNA involved in liver pathophysiology including two lncRNA involved in lipid metabolism *HULC* and *lincRNA-DYNLRB2–2* and Ananthanarayanan [35] reports three other lncRNAs involved in triglyceride, cholesterol and bile acid homeostasis: *lnc-HC*, *lncLSTR*, *APOA1-AS*. In 2017, Zhao et al. [36] present a review on lncRNA involved in liver metabolism and cholestatic liver disease in which *lncLSTR*, *Lnc18q22.2*, *SRA1*, *HULC*, *MALAT1*, *lncHR1* were related to the lipid metabolism and *lnc-HC*, *APOA1-AS*, *H19*, *MEG3*, *lincRNA-DYNLRB2–2*, *LeXis* involved in cholestatic liver pathologies. More recently, in 2018, Van Solingen et al. [17] present a review covering the 12 lncRNA previously described by Zhao et al. enriched with *RP5-833A20.1* renamed *NFIA-AS1* and *MeXis* a new lncRNA discovered in early 2018 by the team of Tontonoz [37]. Likewise recently, Zeng et al. [38] added eight other lncRNAs: Gm16551, *SPRY4-IT1*, *APOA4-AS*, *LINK-A*, *RP1-13D10.2*, *E330013P06* (named *CARMN* in databases), *LOC100506036* (named *CNNM3-DT* in databases) and *SNHG14*. All these reviews report a total of 27 lncRNA involved in lipid metabolism. An extensive literature analysis allowed us to add 33 new lncRNAs, bringing the total to 60 lncRNAs. These extra lncRNAs include one identified in 2015 not mentioned in the aforementioned studies, it was the lncRNA *AT115872* described with the lncRNA *AT102202* (more largely cited in the literature). The first acts at distance on the expression of *ACAT2*, that encodes a key enzyme for the absorption of dietary cholesterol, while the second acts locally on the *HMGCR* gene, which encodes a key enzyme for cholesterol anabolism [39]. Other genes have been described between 2016 and today: 8 in 2016, 15 in 2017 and 15 in 2018. These 60 genes potentially involved in lipid metabolism are listed in Table 1. Most of these genes have been identified in human (34) and mouse (19) with two in both species, the others have been described in rat (1) or livestock species as pig (5) and chicken (4).

**Table 1:** The 60 genes involved in lipid metabolism and their associated publication

Name in article	Database name (feature)	Normalized name	Sp.	Experiment	Tissue/Cell type	Action	Ref.
ANRIL	CDKN2B-AS1 (h: 28tr; 3837 bp; 19ex)	ANRIL	h	Co-effects (6 yr-adiposity)	Umbilical cord	↗lnc & ↗fat mass	[40]
APOA1-AS	APOA1-AS (h: 2tr; 956 bp; 3ex)	APOA1-AS	h	KD (lnc)	HepG2	↗APO gene cluster: APOA1, APOC3, APOA4	[41]
APOA4-AS	Gm10680 (m: 1tr; 702 bp; 2ex)	APOA4-AS^a^	m	KD (lnc)	Liver	↘APOA4 & ↘TG, Chol.	[42]
FLRL5			m	Co-effects (NAFLD)	Liver	↗lnc, FADS2, ↘FABP5, LPL, ACMSD,	[43]
AT102202	–	HMGCR	h	KD (lnc)	HepG2	↗HMGCR	[39]
AT115872	SOD2-OT1 (h: 1tr; 1718 bp; 2ex)	SOD2-OT1^a^	h	KD (lnc)	HepG2	↘HMGCR	[39]
Blnc1	–	Blnc1^a^	m	Ov. (lnc)	Primary hepatocytes	↗SREBP1c, FASN, SCD, DGAT2, FABP4, ABCA1, ABCG5, LPCAT3	[44]
CASIMO1	SMIM22^b^	SMIM22^a^	h	Ov. (lnc)	MCF-7	↗Lipid droplet formation	[45]
CRNDE	CRNDE (h: 24tr; 6325 bp; 2ex)	CRNDE	h	KD (lnc)	HCT116, HT29	↘FASN, DGKA, CDS1, PLCB3, PLCG1, ↗ACADVL, ACOT9, PI4KB	[46]
ORat7			c	Co-effects (Se-deficient diet)	Vein	↗lnc, ↘ACADS	[47]
E330013P06	Carmn (m: 2tr; 1778 bp; 3ex)	CARMN	m	Ov. (lnc)	RAW 264.7	↗lipid uptake	[48]
FLRL3	Gm11832 (m: 1tr; 432 bp; 3ex)	RAD54B-AS1^a^	m	Co-effects (NAFLD)	Liver	↗lnc, FADS2, ↘FABP5, LPL, ACMSD,	[43]
FLRL8	1700067K01Rik^b^	-^a^	m	Co-effects (NAFLD)	Liver	↗lnc, FADS2, ↘FABP5, LPL, ACMSD,	[43]
Gm16551	Gm16551 (m: 5tr; 3162 bp; 3ex)	LINCxxxx^a^	m	KD (lnc)	Liver	↗ACLY, FASN, SCD & ↗TG	[49]
H19	H19 (m: 7tr; 2286 bp; 5ex)	H19	m	Co-effects (FA)	Liver	↗lnc, PTBP1	[50]
HAGLR	HAGLR (h: 17tr; 4095; 3ex)	HAGLR^a^	h	KD (lnc)	NSCLC cells	↘FASN & ↘FA	[51]
HOTAIR	HOTAIR (h: 5tr; 2421 bp; 7ex)	HOTAIR^a^	h	KD (lnc)	CNE2, 5-8F	↘FASN & ↘FA	[52]
HOXC-AS1	HOXC-AS1 (h: 2tr; 548 bp; 2ex)	HOXC-AS1^a^	h	Ov. (lnc)	THP-1	↗HOXC6 & ↘Chol.	[53]
HULC	–	HULC	h	Co-effects (HCC)	HepG2	↗lnc, ACSL1, PPARA & ↗TG, Chol.	[54]
LeXis	4930412L05Rik (m: 2tr; 1241 bp; 8ex)	LeXis	m	KO, KD, Ov. (lnc)	Liver	↘CYP51A1, FDPS, MVK, MVD, SQLE, IDI1, LSS, PMVK & ↘Chol.	[55]
LINC01138	LINC01138 (h: 14tr; 2212 bp; 4ex)	LINC01138^a^	h	KD, Ov. (lnc)	Renal cell carcinoma	↗SREBP1 activity & ↗lipid desaturation	[56]
linc-ADAL	LINCADL (h: 1tr; 521 bp; 2ex)	LINCADL^a^	h	KD (lnc)	ASC	↘PPARG, CEBPA, SREBF1, FASN, ELOVL6, ATGL & ↘TG	[57]
lincRNA-DYNLRB2–2	LINC01228 (h:1tr; 623 bp; 2ex)	LINC01228	h	Co-effects (Ox-LDL)	THP-1	↗lnc, GPR119, ABCA1 & ↘Chol., ↗Efflux	[58]
LINK-A	LINC01139 (h: 7tr; 1579; 2ex)	LINK-A^a^	h	Co-effects (lipid-binding)	TNBC	↗lnc with highest lipid enrichment	[59]
LISPR1	–	S1PR1-DT^a^	h	KD (lnc)	HUVEC	↗S1PR1	[60]
lnc_DHCR24	DHCR24-DT (h: 4tr; 440 bp; 2ex)^c^	DHCR24-DT^a^	c	Co-effects (fat line)	Liver	↗lnc, DHCR24	[20]
lnc18q22.2	LIVAR (h: 1tr; 384 bp; 2ex)	LIVAR	h	Co-effects (NASH)	Liver	↗lnc, anti-apoptotic genes	[61]
lncACACA	–	LINCxxxx^a^	h	Co-effects (LXR agonist)	THP-1	↗lnc	[62]
lncARSR	LNCARSR ^d^ (h: 10tr; 2932 bp;2ex)	lncARSR^a^	m	Ov. (lnc)	Liver	↗SREBP1c, FASN, ACC1, SCD, ↘CPT1A	[63]
			m	Ov. (lnc)	Liver	↗HMGCR, HMGCS, SQLE, ↘CYP7A1 & ↗Chol.	[64]
lncFASN	LINC01970 (h: 1tr; 1810 bp; 2ex)	LINC01970^a^	h	Co-effects (LXR agonist)	THP-1	↗lnc	[62]
lnc-HC	–	lnc-HC	r	KD, Ov. (lnc)	Liver, CBRH-7919	↘CYP7A1, ABCA1 & ↘TG, Chol.	[65]
lncHR1	AC023161.1 ^d^ (h: 1tr; 420 bp; 2ex)	lncHR1	m	Ov. (lnc)	Liver	↘SREBP1c, FASN, ACACA & ↘TG	[66]
lnc-KDM5D-4	–	LINCxxxx^a^	h	KD (lnc)	HepG2	↗LPIN2 & ↗Lipid droplet formation	[67]
lnc-leptin	–	lnc-leptin^a^	m	KD (lnc)	Primary adipocyte	↘LEP	[68]
lncLSTR	C730036E19Rik (m: 1tr; 1102 bp; 5ex)	lncLSTR	m	KD (lnc)	Liver	↗APOC2, ↘CYP8B1 & ↘TG, Glucose	[69]
lncLTR	NONGGAG001747.2 (c: 1tr; 776 bp; NA)	lncLTR^a^	c	GWAS (serum TG content)	–	SNP in lncLTR locus	[70]
lncSHGL	B4GALT1-AS1 ^d^ (h: 3tr; 3752 bp; 4ex)	lncSHGL^a^	m	KD, Ov. (lnc)	Liver	↗ACACB, ↘FASN, SREBP1 & ↘FA, lipolyse	[71]
lncSREBF1	SMCR2 (h:1tr; 564 bp; 4ex)	SMCR2^a^	h	Co-effects (LXR agonist)	THP-1	↗lnc	[62]
LNMICC	AC009902.2 (h: 2tr; 620 bp; 2ex)	LNMICC^a^	h	KD, Ov. (lnc)	HeLa229	↗ACACA, FASN, FABP5, ↘ACOX1, CPT1A & ↗TG, PL	[72]
LOC100506036	CNNM3-DT (h: 1tr; 415 bp; 2ex)	CNNM3-DT	h	KD (lnc)	Jurkat cells	↘SMPD1, NFAT1	[73]
LOC157273	AC022784.6 (h: 1tr; 559 bp; 1ex)	LINCxxxx^a^	h	GWAS (lipid-traits)	–	↗lnc / SNP in the lncRNA locus	[74]
MALAT1	MALAT1 (h: 17tr; 1519 bp; 2ex)	MALAT1	h	KD (lnc)	HepG2	↗SREBP1c & ↗TG, Chol.	[75]
MEG3	MEG3 (h: 50tr; 4867 bp; 2ex)	MEG3	h,m	Ov. (lnc)	Liver HEK-293 T	↗CYP7A1, CYP8B1, FXR, SREBP1c, ↘SHP	[76]
			m	KD (lnc)	Liver	↘TG	[77]
			m	Co-effects (NAFLD)	Liver	↗lnc, NRF2, ↘miR-136 & ↘serum lipid	[78]
MeXis	AI427809 (m: 4tr; 2033 bp; 2ex)	MeXis	m	KO, Ov. (lnc)	Liver, Macrophage	↗ABCA1 & ↗Chol. efflux	[37]
NEAT1	NEAT1 (h: 9tr; 3341 bp; 2ex)	NEAT1	h	KD (lnc)	THP-1	↘TNFa, ↗CD36, OLR1 & ↗lipid uptake	[79]
			h	KD (lnc)	HCC	↘ATGL, PPARa, ↗miR-124-3p	[80]
OLMALINC	OLMALINC (h: 36tr; 5893 bp; 5ex)	OLMALINC^a^	h	Co-effects (obesity)	Adipose	↘lnc, ↗lipid metabolism genes	[81]
			h	KD (lnc)	HepG2	↗SREBP2-dependent gene, ↘SREBP1 pathway genes	[82]
PLA2G1Bat1	ENSGALG00000041755 (c: 3tr; 2101 bp; 3ex)	LINCxxxx^a^	c	Co-effects (Se-deficient diet)	Vein	↘lnc, ↗PLA2G1B	[47]
PVT1	PVT1 (h:182tr; 1699 bp; 8ex)	PVT1^a^	h	KD (lnc)	U2OS MG-63	↗miR-195, ↘FASN	[83]
RNCR3	Mir124a-hg (m: 4tr; 4103 bp; 4ex)	RNCR3^a^	m	KD (lnc)	Plasma	↗TG, Chol.	[84]
RP1-13D10.2	AL021407.3 (h: 1tr; 486 bp; 1ex)	LINCxxxx^a^	h	Ov. (lnc)	Huh7, HepG2	↗LDLR & ↗LDL, ↘ApoB	[85]
RP5-833A20.1	NFIA-AS1 (h: 7tr; 384 bp; 4ex)	NFIA-AS1	h	KD, Ov. (lnc)	THP-1	↗miR-382, ↘NFIA & ↗Chol.	[86]
SNHG14	Snhg14 (m: 15tr; 6861 bp;1 2ex)	SNHG14	m	KD, Ov. (lnc)	BV-2	↘PLA2G4A	[87]
SNHG16	SNHG16 (h: 13tr; 3607 bp; 3ex)	SNHG16^a^	h	KD (lnc)	HCT119	↘SCD, PCSK9, SQLE, ACLY, INPP5D, HSD17B7	[88]
SPRY4-IT1	SPRY4-AS1 (h: 7tr; 1293 bp; 5ex)	SPRY4-IT1^a^	h	KD (lnc)	HEM-1	↘DGAT2, GPAT3 & ↘Acyl Carnitine, FA, TG	[89]
SRA	Sra1^b^ (m: 2tr; 1299 bp; 4ex)	SRA1	m	KO (lnc)	Liver	↗ATGL	[90]
			m	KO (lnc)	Liver	↘PPARA, PPARG, FABP4, SCD & ↘TG, FA	[91]
TRIBAL	AC091114.1 (h: 2tr; 1272 bp; 3ex)	TRIBAL	h	GWAS (TG)	–	SNP in TRIBAL locus	[92]
uc.372	–	RALGAPA1-AS1^a^	h,m	Ov. (lnc)	Liver, HepG2	↗ACACA, FASN, SCD, CD36 & ↗TG, Chol.	[93]
XLOC_011279	–	LINCxxxx^a^	p	Co-effects (fat line)	Adipose	↗lnc, LPIN1	[94]
XLOC_013639	–	LINCxxxx^a^	p	Co-effects (fat line)	Adipose	↗lnc, SCD	[94]
XLOC_014379	–	NF1-IT1^a^	p	Co-effects (fat line)	Adipose	↗lnc, SCD	[94]
XLOC_019518	–	RNF7-DT^a^	p	Co-effects (fat line)	Adipose	↗lnc, SCD	[94]
XLOC_064871	–	LINCxxxx^a^	p	Co-effects (fat line)	Adipose	↗lnc, TRIB3	[94]

^**a**^genes that not described in previous reviews dedicated to lncRNA in lipid metabolism. **Database name (feature):** name used in the Ensembl database for human (h), mouse (m) or chicken(c) gene database depending on the species in which the lncRNA has been studied (see column “sp”), the database was NONCODE for lncLTR; between brackets, the following features are provided: transcript number; the size (in bp) and the exon number (ex) indicated only for the transcript having the highest size and noted in the database ‘genecode basic’ and/or ‘TSL1 or TSL2’ (indexes giving the transcript support level)); ^b^: lncRNA with a double “protein coding-lncRNA” classification (see result section); ^c^: human name for lncRNA discovered in chicken and for which a non ambigous 1-to-1 orthologue was found; ^d^: human name if no mouse name was found in Ensembl database. Normalized name: new names according to the HUGO gene nomenclature committee [32]. **“Sp.”** column mentions the species studied: “h”, “m”, “r”, “p” and “c” for human, mouse, rat, pig and chicken, respectively. The **“Experiment”** column refers to three type of experiments: **1**. a direct or indirect causative effect of the lnc on the lipid metabolism through either an invalidation of the lncRNA by knockout (KO) or knockdown (KD) or an overexpression (Ov.) performed in vitro in cells or in vivo in mice; in the “action” column, we have provided the effects of the lnc overexpression when there were Ov and KD/KO experiments; **2**. a “Co-effect “refers to a modulation of the quantity of the lncRNA in parallel with the quantity of transcripts or metabolites known to be involved in lipid metabolism; this co-modulation being induced in response to a particular factor (given between brackets) as disease, genotype/line, diet or molecule known to modulate lipid metabolism; **3**. a” genome-wide association study” analysis (GWAS). The information in the “**Tissue/cell type” and “Action”** columns correspond to the experiment

### Mechanisms of action of lncRNAs

The demonstration of a link between the lncRNA of interest and lipid metabolism can be variable from a publication to another (Table 1). For 38 of the 60 genes, a direct or indirect causative effect of the lncRNA on the lipid metabolism is given in response to an invalidation and/or an overexpression of the lncRNA. Such experiments were conducted in human (22) or murine (4) cell cultures or through in vivo experiments, by injection of viral vector in the tail of mice (11) or more rarely KO mice (3 with *LeXis*, *MeXis* and *SRA1* lncRNAs). Out of these 38 studies, 25 go further to partially or totally decipher the action mechanism (Table 2). For 20 of the 60 genes, the link with lipid metabolism was only based on a co-expression between the lncRNA and one (several) transcript(s) or metabolite(s) related to the lipids in response to a disease (10), a genotype (6) or a molecule (8) known to act more or less specifically on lipid metabolism. Finally, 3 studies reported a link with lipid metabolism by GWAS analysis between genetic markers within or close to the lncRNA and a phenotype associated to lipid metabolism suggesting that the lncRNA is a potential causal gene for the phenotype variation.

**Table 2:** Mechanisms of action reported for 25 lncRNAs

Name	Sp.	LncRNA action	Ref.
APOA1-AS	h	Histone mark modification via LSD1 et SUZ12 (PRC2)	[41]
APOA4-AS	m	Complex lnc:HuR et APOA4 mRNA	[42]
Blnc1^a^	m	Complex lnc:hnRNPU / Complex lnc:EDF1, lnc:LXRa	[95]
CASIMO1^a^	h	Small protein CASIMO1 interacts with SQLE protein	[45]
H19	m	Complex lnc:PTBP1 which targets SREBP1c mRNA and protein stability and transcriptional activity	[50]
LeXis	m	Complex lnc:RALY (hnRNP) which targets SREBP2, HMGCR, CYP51A1, FDPS promoters	[55]
LINC01138	h	Comple lnc:PRMT5 which targets SREBP1 in order to regulate its arginine methylation and to stabilize SREBP1 mRNA	[56]
linc-ADAL^a^	h	Complex lnc:IGF2BP2 and hnRNPU	[57]
LINK-A^a^	h	Complex lnc:PIP3 increases interaction with Akt	[59]
lnc-HC	r	Complex lnc:hnRNPA2B1 which targets CYP7A1/ABCA1 mRNAs	[65]
Lnc-leptin^a^	m	Loop between LEP and its enhancer (near lnc:leptin locus)	[68]
lncLGR^a^	m	Complex lnc:hnRNPL which targets GCK promoter	[96]
lncLSTR	m	Complex lnc:TDP-43 which targets CYP8B1 promoter	[69]
lncSHGL^a^	m	Complex lnc:hnRNPA1 which targets CALM mRNA to increase its translation	[71]
LNMICC^a^	h	Complex lnc:NPM1 which targets FABP5 promoter	[72]
MALAT1	h	Complex lnc:SREBP1c which stabilizes nuclear SREBP1c protein	[75]
MEG3	h,m	Complex lnc:PTBP1 which targets SHP mRNA for its decay	[76]
MeXis	m	Complex lnc:DDX17 which targets ABCA1 promoter	[37]
NEAT1	h	Complex lnc:NONO:CD36 mRNA	[79]
	h	ceRNA (miR-124-3p which targets ATGL)	[80]
PVT1^a^	h	ceRNA (miR-195 which targets FASN)	[83]
RALGAPA1-AS1^a^	h,m	Stop maturation of pri-miRNA (pri-miR-195/pri-miR-4668)	[93]
S1PR1-DT^a^	h	Complex lnc:ZNF354C which inhibits its repressive activity	[60]
SNHG14	m	ceRNA (miR-145-5p which targets PLA2G4A)	[87]
SNHG16^a^	h	ceRNA (multiple miRNA which target SCD) & Complex lnc:HuR	[88]
SPRY4-IT1^a^	h	Complex lnc:Lipin2	[89]

^a^genes not described in previous reviews

**Fig. 1:**
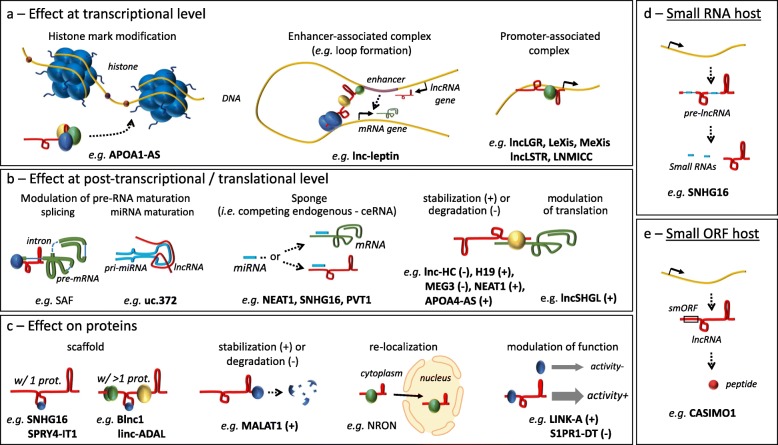
The different mechanisms of action of lncRNAs. **a** Mechanisms with effect at transcriptional level, **b** at post-transcriptional level and (**c**) on proteins. **d** LncRNAs with a role as small noncoding RNA host. **e** LncRNAs with translational activity through a small ORF. In red, lncRNA; in green, mRNA; in blue, miRNA; the green, yellow and blue oval spheres are proteins. The genes in bold are those mentioned in this review, the others are examples from research fields other than lipid metabolism: SAF^66^ and NRON [97]

All the 60 lncRNAs we suggested as involved in lipid metabolism cover most of the types of action described so far in the literature for the noncoding genes. As shown in Fig. 1, these lncRNAs may function as regulators of the transcription by acting at the DNA level (Fig. 1a), of the post-transcription and translation by acting at the RNA level (Fig. 1b) and finally of the post-translation by acting at the protein level (Fig. 1c). Concerning the underlying biochemical mechanisms, most of them are based on lncRNA-RNA or lncRNA-protein(s) interactions. RNA immunoprecipitation and pull-down assays [98] have revealed a vast range of interactions between lncRNAs and proteins, proteins that sometimes interact with other RNAs. Such interactions constitute real scaffolds that can inhibit or activate different biological processes. At the transcriptional level (Fig. 1a), different studies showed an action of lncRNAs on the promoters of genes involved in lipid metabolism. For example, it is the case of *LeXis* as reported in a very comprehensive study conducted by the Tontonoz’s lab [55] (Fig. 1a, right part): first, *LeXis* was observed as the most up-regulated lncRNA in mouse primary hepatocytes when treated with GW3965, an agonist to the liver X receptor (LXR) that mediates cellular and systemic cholesterol homeostasis and in particular inhibits cholesterol biosynthesis. The existence of a response element to LXR was then demonstrated in the *LeXis* promoter using luciferase reporter gene experiment and ChIP-qPCR. Using overexpression of *LeXis* by adenovirus injection or knockdown experiments in mouse, the authors show that *LeXis* decreased cholesterol and HDL and decreased the expression of genes involved in the cholesterol biosynthesis pathway. LeXis^−/−^ mice also showed an increase in hepatic cholesterol. Present in the nucleus, *LeXis* is suspected to interact on gene transcription by modifying protein recruitment on chromatin. Tontonoz’s lab then demonstrates, using ChIRP/SM and ChIP experiments, a binding of *LeXis* on the heterogeneous nuclear ribonucleoproteins (hnRNP) RALY, suspected to be a potential transcriptional co-factor. Its knockdown is responsible for a decrease in cholesterol levels as well as genic expression in cholesterol biosynthetic pathway. The authors show, using knockdown and ChIP-qPCR experiments that RALY binds the promoter of different cholesterologenic genes and activates their expression, activation affected by *LeXis* through the modulation of RALY DNA-binding [55].

Other studies have shown an action of lncRNAs on the transcription. For example, *APOA1-AS* seems to inhibit the transcription of the *APO* gene cluster *(APOA1, APOC3, APOA4, APOA5)* that codes for protein components of lipoproteins, by DNA compaction through epigenetics mark modulation [41] (Fig. 1a, left part). This mechanism seems to require the recruitment of the LSD1 protein known to induce gene silencing through the removal of active methyl marks, and of the SUZ12 protein, a key component of the polycomb recessive complex (PRC2) known to mediate chromatin silencing through H3K27 trimethylation [41]. Indeed, the *APOA1-AS* depletion in HepG2 cells increased the active histone H3K4me3 marks at the *APOA1* promoter in parallel to a significant decrease of LSD1 occupancy. *APOA1-AS* depletion also decreased the repressive histone H3K27me3 marks at the *APOA1* promoter that coincided with a marked reduction of SUZ12 occupancy in this region [41]. A second example of lncRNA action on DNA compaction is Lnc-leptin [68] (Fig. 1a, middle part). By using chromatin conformation capture experiments, a direct interaction was detected between *lnc-leptin* and the *LEP* (*leptin*) gene, which codes for a major adipokine secreted by white adipocytes and functioning as an energy sensor to regulate energy homeostasis. This “*lnc-leptin* – *leptin* promoter” interaction occurred at the enhancer region of *LEP* and it was diminished upon *lnc-leptin* knockdown in mature adipocytes.

At the post-transcriptional level, some lncRNAs seem to play a role in the maturation of RNAs such as lncRNA *uc.372* (Fig. 1b, left part) which prevents the maturation of a pri-miRNA by camouflaging the area targeted by the Drosha protein [93]. On mature RNAs, the so called “competing endogenous RNA (ceRNA)” role of lncRNAs resembles that of a sponge for small RNAs regulating the mRNA target of small RNAs (Fig. 1b, middle part). Among the lncRNAs involved in lipid metabolism, *NEAT1* [80], *SNHG16* [88] and *PVT1* [83] are endogenous competitors of *ATGL*, *SCD* and *FASN* transcripts, respectively. Sometimes, lncRNA-protein complexes target a mRNA and thus regulate its stability (*lnc-HC* [65], *H19* [50], *MEG3* [76], *NEAT1* [79], *APOA4-AS* [42]) or its translation (*lncSHGL* [71]) (Fig. 1b, right part). LncRNAs can bind via their three-dimensional conformation, involving one or more proteins in the structure (Fig. 1c). They can bind via their sequence RNA of hnRNP such as *SPRY4-IT1* [89] (Fig. 1c, left part). They can also form protein scaffolds such as *Blnc1* which associates with EDF1, LXRα and hnRNPU [95] or *linc-ADAL* which associates with IGF2BP2 and hnRNPU [57] (Fig. 1c, left part). These protein scaffolds do not yet have a well-defined mechanism of action. Some publications have gone further by highlighting the interest of this complex. For example, lncRNA protein complexes can modulate the half-life of the proteins involved in the complex, such as *MALAT1* which binds to SREBP1c in order to stabilize it [75] (Fig. 1c, middle part), or they can modulate its function, such as *LINK-A* which binds to PIP3 and intensifies its interaction with Akt [59] (Fig. 1c, right part) or modify its cell location as illustrated by NRON [97]. Another role of lncRNA [99] is that of hosting small RNAs (Fig. 1d). *SNHG16* [88] illustrates very well this role because it hosts 3 small nucleolar RNAs (snoRNA) *SNORD1A*, *SNORD1B* and *SNORD1C* which are positioned within the introns in the sense direction of the lncRNA, thus benefiting from the co-transcription with the host lncRNA. Finally, lncRNAs can also host small ORFs (Fig. 1e) allowing the translation of small peptides. *CASIMO1* is a perfect example, lncRNA *CASIMO1* hosts the sequence of a small transmembrane peptide that interacts directly with the SQLE protein and modulates the formation of lipid droplets [45].

### New names proposed for misnamed lncRNAs

**Fig. 2:**
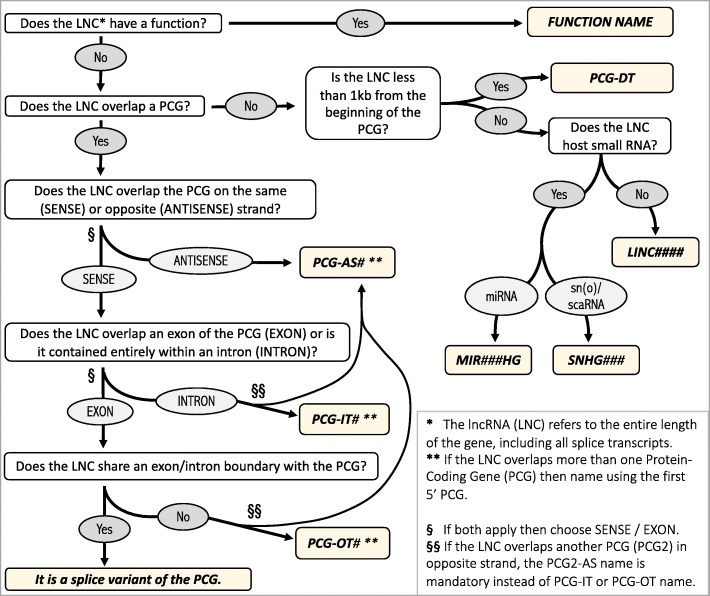
HGNC decision tree for naming lncRNAs according to the Wright’s schema [32], here updated by including divergent lncRNA and lncRNA hosting small noncodingRNA

When a new field is explored, the associated nomenclature requires a certain amount of time to be standardized; this was the case for the nomenclature associated with protein-coding genes and of course it is also the case for the long noncoding genes. When referring to the official HUGO gene nomenclature committee (HGNC), it appears that some lncRNA were not properly named, which can lead to misunderstandings. Indeed, the HGNC lists very precise rules on how to name lncRNA [32]. These can be summarized in seven points (Fig. 2): 1. if the lncRNA function is well described, the lncRNA takes an abbreviated name symbolizing its function, e.g. *LeXis* for liver-expressed LXR-induced sequence, *lncLSTR* for liver-specific triglyceride regulator. In the case of unknown function, the lncRNA takes the symbol gene name of the gene harboring it enriched by a suffix describing its genomic location: 2. the ‘Intronic’ and ‘sense’ lncRNA genes are appended with -IT for Intronic Transcript (e.g. *SPRY4-IT1*); 3. the ‘sense’ and overlapping a protein-coding gene lncRNA gene are appended with the suffix -OT for Overlapping Transcript (e.g. *SOD2-OT1*); 4. the ‘Antisense’ lncRNA gene are appended with the suffix -AS for Antisense (e.g. *APOA1-AS*, *NFIA-AS1*). 5. Close intergenic divergent lncRNA (< 1 kb) transcribed in the opposite direction to nearby protein-coding genes takes the gene symbol name appended with the suffix -DT for Divergent Transcript (e.g. *DHCR24-DT*). 6. Other intergenic lncRNAs take the name LINC followed by a number assigned by HGNC committee (for human genes). 7. An exception exists for lncRNAs hosting small noncoding RNAs that take the name of the small hosted RNA appended with the suffix HG for Host Gene (e.g. *SNHG16*). Finally a long noncoding transcript that has common splice junctions with protein-coding transcripts is considered as an additional isoform and therefore belongs to this protein-coding gene [32]. In spite of this approved nomenclature guidance, some lncRNAs are still misnamed. For example the lncRNAs described by Tristán-Flores et al. [62] have been misnamed in *lncACACA*, *lncFASN* and *lncSREBF1*, although they are neither sense nor antisense of the *ACACA, FASN* and *SREBP1* genes. The lncRNA depletion by KO experiments affected the expression levels of the three genes, which again does not allow to use these names.

We propose to rename some lncRNAs according to the HGNC rules whatever the species (Table 1, column 3). Seven lncRNA have been renamed according to the nearby protein-coding gene: *lnc_DHCR24* [20] in *DHCR24-DT*, *AT115872* [39] in *SOD2-OT1*, *XLOC_014379* [94] in *NF1-IT1*, *FLRL3* [43] in *RAD54B-AS1*, *XLOC_019518* [94] in *RNF7-DT*. We have also renamed two other genes: *LISPR1* (long intergenic noncoding RNA antisense to *S1PR1*) [60] in *S1PR1-DT*, and *uc.372* (ultra-conserved 372) [93] which has a generic name and should be rather called *RALGAPA1-AS1*. We have restored the official and standardized names, *LINC01970* and *SMCR2* (*Smith-Magenis syndrome Chromosome Region, candidate 2*), for the two *lncFASN* and *lncSREBF1* genes [62] respectively. All other *XLOC_013639*, *XLOC_011279*, *XLOC_064871* [94], *lncACACA* [62], *LOC157273* [74], *Gm16551* [49], *PLA2G1Bat1* [47] and *lnc-KDM5D-4* [67] genes should have a standard name of the type *LINC#####*. Finally, the lncRNA *AT102202* has several common splice junctions with the *HMGCR* protein-coding gene and should therefore be considered as a *HMGCR* isoform rather than a lncRNA new gene. Note that the knockdown of this isoform in HepG2 cells was described by Liu et al. to increase the expression level of the gene encoding HMGCR due to the presence of several *HMGCR* isoforms with different expression patterns [39]; although the authors missed to specify which isoform was targeted by siRNAs and which one was up-regulated (see Additional file 1). According to this figure, the knockdown of the *AT102202* isoform should lead to an absence of HMGCR functional protein.

In some cases, a gene is named with several aliases between species. This is the case for *lncSHGL* in mouse known as *B4GALT1-AS1* in human which were both mentioned in Wang et al. [71]. Naming problems also exist within the same species. For example, the NCBI mouse gene named Gm30838 by the MGI (Mouse Genome Informatics), is double named in Lo et al. *lnc-ORIA9* and *lnc-leptin* [68]. Similarly, the human *ENSG00000266304* lincRNA gene, firstly known as *RP11-484 N16.1*, was named *lnc18q22.2* by Atanasovska et al. [61] and finally officially renamed *LIVAR*. Due to their recent studies, we found some genes in databases with an “ID” name instead of its common names used in the literature (e.g. *AC023161.1* instead of *lncHR1* [66]). Other problems are due to some articles that use lncRNA gene names without referring to any database: the most classical examples are XLOC/TCONS names reflecting experiment-dependent naming from Cufflinks (e.g. pig new genes in Huang et al. [94]) but other names exist such as *AT102202* [39] which does not refer to any easily detectable source because it is a contraction of the NONCODE ID *NONHSAT102202*.

### Detailed examination of model architecture

**Fig. 3:**
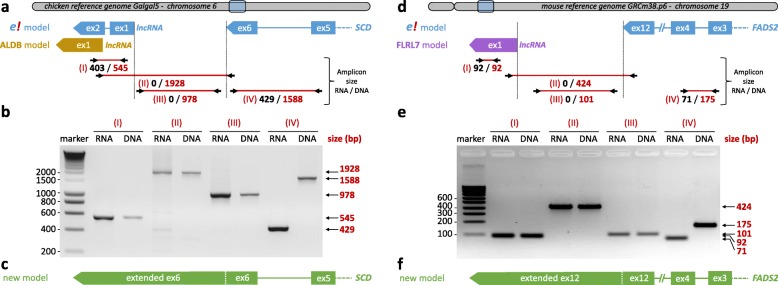
Verification of the *ALDBGALG00000000505049* and *FLRL7* gene models with their neighboring *SCD* and *FADS2* genes in chicken and mouse. **a**, **d** Model of protein-coding genes are from Ensembl v.92 and models of lncRNA are from the article of Fan et al. [101] that used ALDB v1.0, a lncRNA database (**a** for *ALDBGALG00000000505049*) and from the article of Chen et al. [43] that used their own models (**d** for *FLRL7*). Primers (black arrows) were design in order to amplify a fragment (red line) specific of lncRNA gene (I), of coding-protein gene (IV), of intergenic region (III) and of a fragment linking both genes (II). The expected sizes have been specified (black for RNA; red for DNA) according to the models. **b**, **e** Electrophoretic gels with the lengths of the amplicons, showing the existence of a unique gene, the lncRNA being an extension of the protein-coding gene. **c**, **f** New experimentally corrected models for the protein-coding gene

It is important to note that the vast majority of RNAs present in the databases are only predictions and that their structure has only very rarely been validated experimentally. A lncRNA model localized close to a protein-coding gene and in a same strand of this latter can be spurious; it is possible to be an extension of the protein-coding gene due to the difficulty of modelling the ends of transcripts [100]. We detected two lncRNA in this case. The *ALDBGALG0000005049* lncRNA is close (~ 1 kb from the *SCD* 3’UTR) and on the same strand of the *SCD* gene (Fig. 3a), that encodes the stearoyl-CoA desaturase enzyme that catalyzes the rate-limiting step in the formation of monounsaturated fatty acids. Fan et al. described in chicken myoblasts that the inactivation of the *ALDBGALG0000005049* gene by siRNA led to an under-expression of the *SCD* gene and a modulation of other genes such as *PPARA, PPARB and PPARG* (Peroxisome Proliferator Activated Receptor) [101] making this lncRNA a potentially important lncRNA in lipid metabolism. However, we propose here to verify the existence of the *ALDBGALG0000005049* gene using chicken liver cDNA treated by DNAse using genomic DNA as control. The amplification of the intergenic region “SCD-ALDBGALG0000005049”, confirmed by sequencing, clearly shows the existence of a transcript that overlaps the two genes (Fig. 3b), demonstrating that *ALDBGALG0000005049* and *SCD* are in fact a single gene (Fig. 3c). We report another case, the lncRNA *FLRL7*, located very close (150 nt from the *FADS2* 3’UTR) and on the same strand of the *FADS2* (fatty acid desaturase 2) protein-coding gene (Fig. 3d) and whose expressions were both up-regulated in the liver of NAFLD mouse according to Chen et al. [43]. We demonstrated using mouse liver cDNA treated by DNAse that *FLRL7* and *FADS2* genes are a unique gene (Fig. 3e), thus extending the 3′ UTR end of the *FADS2* gene (Fig. 3f).

### Conservation between phylogenetically distant species

Gene conservation during evolution, especially between phylogenetically distant species suggests an important functional role for these genes. Conservation of function of lncRNA across species was tested in some studies, showing a function retained despite a minor sequences conservation [29]. However the task remains difficult because of the poor conservation of lncRNA sequences between phylogenetically distant species as opposed to protein-coding genes, suggesting a more rapid evolution of lncRNA during evolution [14, 20, 27]. Therefore, we propose here to carry out orthologous lncRNA searches between phylogenetically distant species (chicken and mammals) using an approach based on the genome synteny as already presented in Muret et al. [20] and Foissac et al. [28] (see Materials & Methods). Even if this manual approach is time-consuming, we investigated the whole 60 long noncoding genes involved in lipid metabolism.

Out of the 60 lncRNA genes mentioned in the Table 1, five genes satisfy the aforementioned criteria between chicken and man (or mouse) and can therefore be considered as orthologous between the species analyzed: there are *CRNDE*, *DHCR24-DT*, *NFIA-AS1*, *PVT1* and *SRA1*. Their roles are described in more detail below. We can note that 5 other lncRNAs are potentially orthologous since 1. they correspond positionally to several lncRNAs in the other species or 2. the gene environment has slightly evolved, e.g. a new coding gene appear in locus or the distance between genes are different between species. These lncRNAs are *lncACACA*, *DYNLRB2–2*, *MeXis*, *PLA2G1Bat1* and *TRIBAL*. Table 3 shows the genomic positions in human, mouse and chicken of these 10 lncRNA. These lncRNAs potentially play important roles in lipid metabolism since they act on the expression of key genes of this metabolism as key enzyme genes (*FASN*, *SCD*, *ACADS*, *ACADVL*, *ATGL*), lipid transport genes (*ABCA1*, *FABP4*) or lipid metabolism transcription factor genes (*PPARA*, *PPARG*).

**Table 3:** The 10 lncRNAs potentially conserved between human/mouse and chicken genomes

LncRNA	Hsa.ID	Hsa.position	Mmu.ID	Mmu.position	Gga.ID	Gga.position
CRNDE	**ENSG00000245694**	**16:54845189–54,929,189(−)**	ENSMUSG00000031736	8:92326033–92,356,120(−)	XLOC_003575 [47]	11:4285705–4,316,300(+)
DHCR24-DT	**ENSG00000233203**	**1:54887563–54,888,850(+)**	–	–	ENSGALG00000035342	8:25343117–25,344,661(+)
PVT1	**ENSG00000249859**	**8:127794533–128,101,253(+)**	ENSMUSG00000097039	15:62037986–62,378,096(+)	ENSGALG00000033453	2:139905946–139,947,148(+)
NFIA-AS1	**ENSG00000237853**	**1:61248945–61,253,510(−)**	RefSeq:102637398	4:98018556–98,024,773(−)	NONGGAG010741	8:27269736–27,278,903(−)
SRA1	**ENSG00000213523**	**5:140537340–140,558,252(−)**	ENSMUSG00000006050	18:36666681–36,679,366(−)	ENSGALG00000040453	13:1649170–1,650,955(−)
DYNLRB2–2	**ENSG00000261082**	**16:79798050–79,827,150(−)**	ENSMUSG00000110620	8:115971635–115,972,071(−)	Mult. ALDB Models	~ 11:14928493–15,189,801(−)
lncACACA	**Unknown**	**17:36810109–36,810,324(−)**	–	–	Mult Ens. Models	~ 19:8345185–8,375,056(−)
MeXis	TCONS_00016111 [55]	~ 9:105092000–105,108,500(+)	**ENSMUSG00000086712**	**4:53261356–53,270,232(−)**	Mult. ALDB Models	~Z:54833466–54,863,390(−)
PLA2G1Bat1	–	–	ENSMUSG00000087292	5:115359078–115,422,863(+)	**ENSGALG00000041755**	**15:9358431–9,366,570(+)**
TRIBAL	**ENSG00000253111**	**8:125466939–125,541,373(+)**	–	–	Mult. ManyDB Models	~ 2:138972973–138,996,132(+)

In bold: species where genes were firstly identified. Other: species where the “orthologous” gene was manually found. “Hsa”, “Mmu” and “Gga” refer to *Homo sapiens*, *Mus musculus* and *Gallus gallus*. “ID” is the Ensembl, NCBI, NONCODE or Cufflinks provisional ID (with article reference in brackets). Position refer to the chromosome position in GRCh38.p12 human assembly, GRCm38.p6 mouse assembly and Gallus_gallus-5.0 chicken assembly

*CRNDE* (ColoRectal Neoplasia Differentially Expressed) is a lncRNA divergent with respect to the *IRX5* gene, at 2 kb and 1.2 kb in human and chicken, respectively. In chicken, it has been described as *ORat7* [47] (ALDBGALT0000001763). Composed of 6 to 9 exons in chicken and 6 exons in human, *CRNDE* is mainly noncoding but some isoforms are known in human to code for small proteins [102]. In human, *CRNDE* knockdown by siRNA in colorectal cancer cell lines leads to an under-expression of *FASN*, and an overexpression of *ACADVL* and *ACOT9*, two genes involved in lipid catabolism [46].

*Lnc*_*DHCR24*, renamed in *DHCR24*-*DT* (Table 1), is a lncRNA divergent with respect to the *DHCR24* gene coding for a key enzyme of the cholesterol synthesis. We have modelled this transcript for the first time in 2017 in chicken and located a possible orthologue in human [20]. The intergenic region is very small, i.e., 200 bp in human and 300 bp in chicken [20]. In a previous study, we have suggested that these two genes are likely to be co-regulated by a bi-directional promoter, because of their high hepatic co-expression in several chicken lines (layers and broilers) analysed at different ages (young and, adult stage) [20].

*NFIA-AS1* is an intronic antisense lncRNA of the *NFIA* gene (Nuclear Factor I A). Composed of 4 to 7 exons, it is found on the second intron of *NFIA* gene in human as well as in chicken. It negatively regulates in THP-1 cells the expression of the *NFIA* gene by a negative feedback mechanism with the transcription factor NF1A binding the promoter of *NFIA-AS1* to activate it [86]. LncRNA *NFIA*-*AS1* (also called *RP5-833A20.1*) is induced by a high level of oxidized and acetylated LDL [86]. These two genes (*NFIA-AS1* and *NFIA)* appear to regulate the homeostasis of cholesterol (LDL, HDL, VLDL) and inflammatory cytokines (IL-1β/6, TNF-α and CRP) in *Apo*^*−/−*^ mice [86].

*PVT1* (Plasmacytoma Variant Translocation 1) is a same strand lncRNA with respect to the *MYC* nearest protein-coding gene. Modelled in the NCBI databases of human, mouse and rat, the two genes are separated by 50-60 kb in these three species. In the chicken, we also found it at the same distance (56 kb) of the *MYC* gene. *PVT1* in human is a competitive endogenous lncRNA by acting as a sponge of MIR-195 in osteosarcoma cells [83]. It upregulates *BCL1* and *BCL2*, two genes playing roles in the control of cell cycle and apoptosis, but also *FASN*, one of the key lipogenic enzymes.

*SRA1*, better known by its first name *SRA* (*Steroid Receptor Activator*), is one of the first lncRNA described in human (1999) [103]. Its functional RNA acts as a steroid receptor coactivator. It can coactivate androgen receptor (AR), estrogen receptors (ERα, ERβ), progesterone receptor (PR), glucocorticoid receptor (GR), thyroid hormone receptor (TR) and retinoic acid receptor (RAR) [104]. As early as 2003, it was demonstrated that its codes for a functional SRAP protein improving the trans-activation of *PPARG* and genes coding for AR and GR [105]. In Human, as in mouse or other species, this double “protein coding-lncRNA” classification exists [106]. In this review, we are only interested in its noncoding isoform. *SRA1* is an antisense exonic lncRNA of the gene coding for *ANKHD1* in human. Currently, we do not know if *SRA1* has noncoding isoforms in chicken but a coding isoform is present in Ensembl database (*ENSGALG00000040453*). Using a mouse SRA1 KO, the noncoding *SRA1* isoform seems to regulate in the liver the expression of genes involved in lipid metabolism as *PPARA, PPARG, FABP4* and *SCD* by a mechanism still unknown [91]. In 2016, the same team showed that in liver, the noncoding *SRA1* isoform positively regulates the expression of *PNPLA2*. *PNPLA2* codes for an enzyme that plays a role in lipid hydrolysis that if under expressed leads to the progressive fatty liver steatosis [90].

**Fig. 4:**
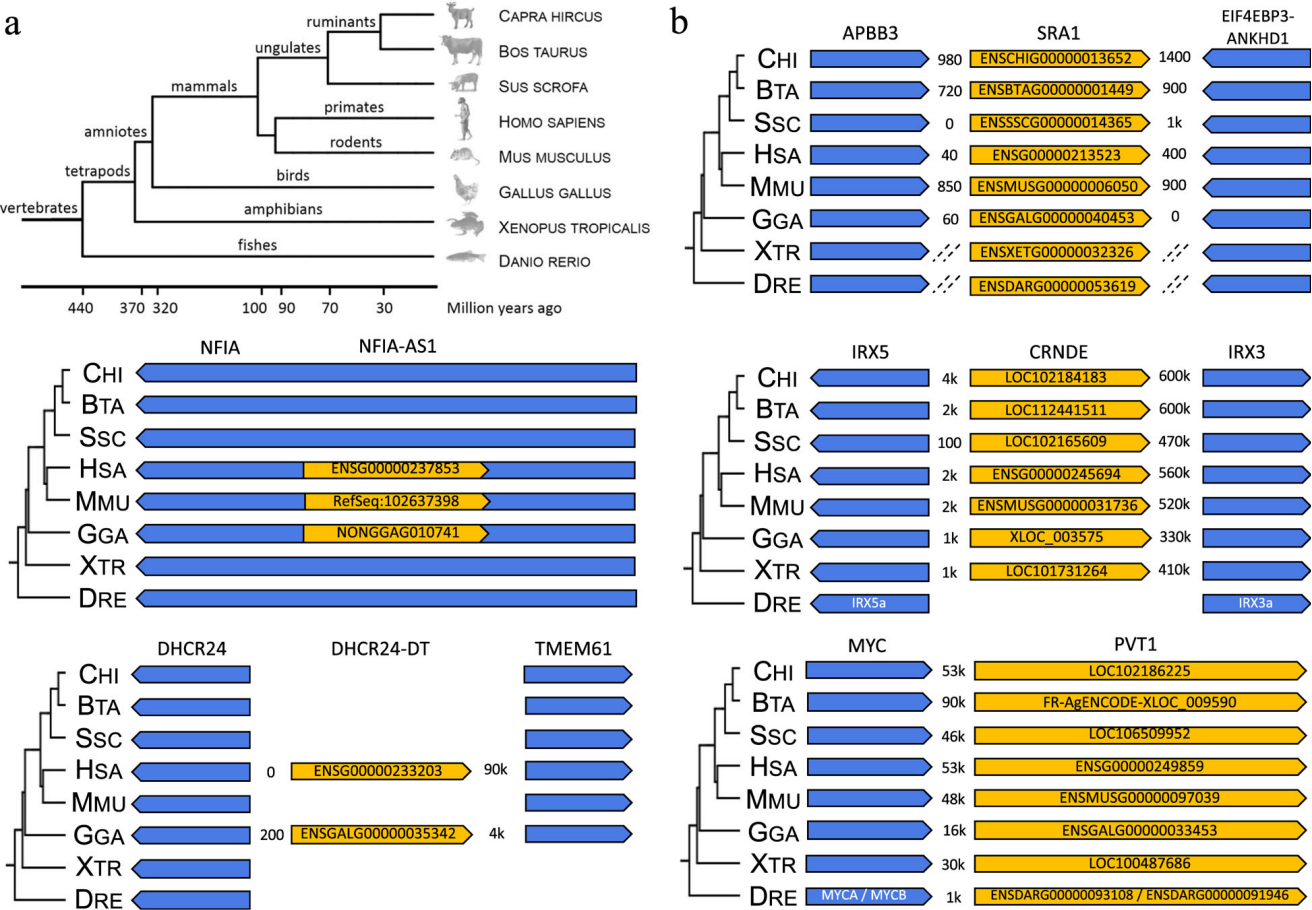
Genomic conservation in 8 species of the five lncRNA previously found as conserved between human and chicken. **a** Tree of genome evolution in vertebrates based on Kumar and Hedges studies [107, 108]. **b** Conservation of the five lncRNA (yellow) through the animal kingdom in relation to their genomic environment: protein-coding gene (blue). The distances between the intergenic entities are in bases

For the previous 5 lncRNA found to be preserved between human and chicken, two model species, xenope and zebrafish, and three livestock species, goat, bovine and pig, have been added to the syntenic conservation analysis to better appreciate the conservation in vertebrates. Note that the model species xenope (370Myr) and zebrafish (440Myr) are more phylogenetically distant from human than the chicken (320Myr) (Fig. 4a). We have selected also cattle and pig because they are economically important livestock species, widely consumed in the world. To study conservation, in addition to the public reference Ensembl and NCBI databases, we used the lncRNA catalogues that we recently published for these species [28]. The Fig. 4b shows the conservation of the 5 lncRNA across the 8 species. In the current state of the genome annotation, two genes *DHCR24-DT* and *NFIA-AS1* are preserved only within some amniotes, despite the fact that the two *DHCR24* and *NFIA* neighboring protein-coding genes were conserved in other vertebrates. Such observations can be due to a poor gene annotation in the species were these two long noncoding genes were not identified. Another hypothesis is a convergent evolution in mammals and birds that could explain why it is present in chicken and not in other mammals that are evolutionary closer to human and mouse. On the other hand, *CRNDE* is found in all tetrapods with robust reliability and the cluster of IRX genes in which *CRNDE* is located is very strongly preserved in vertebrates [109]. Finally, two genes are conserved in all vertebrates: *PVT1* and *SRA1* which are in particular involved in FA biosynthesis through the regulation of two key enzymes FASN and SCD respectively.

**Fig. 5:**
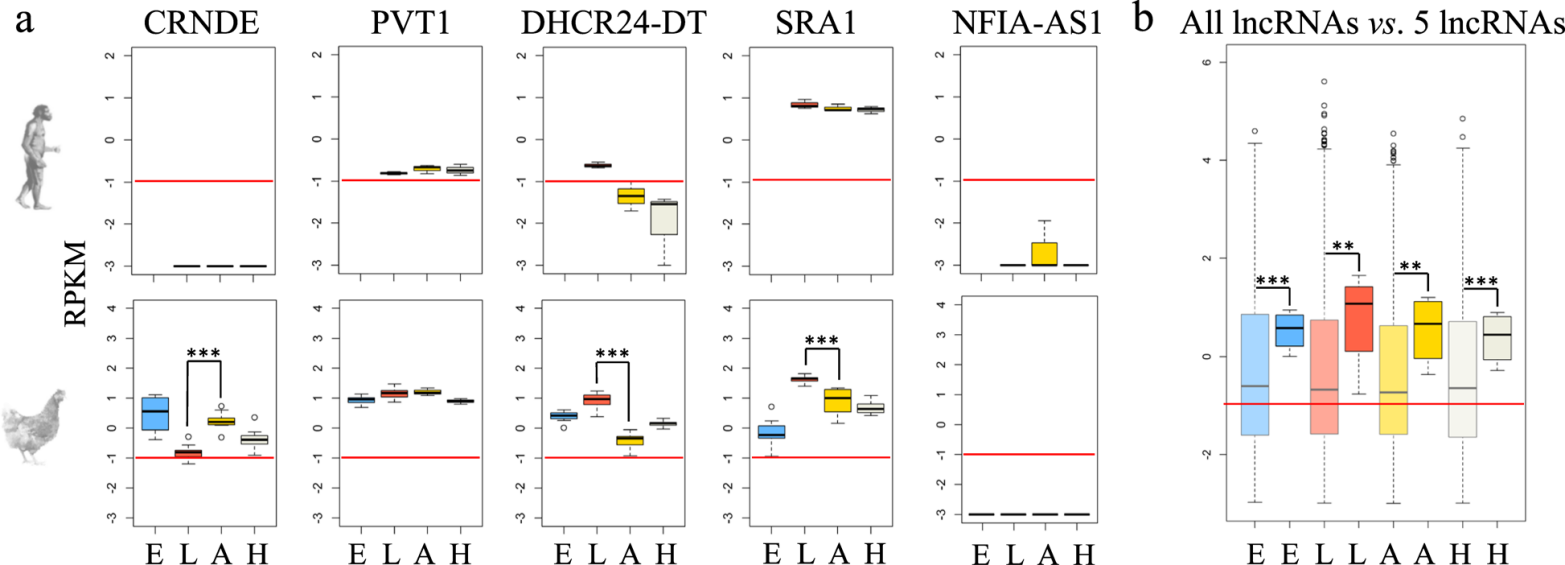
Expression of lncRNA in embryo, liver, adipose tissue and hypothalamus in human and chicken. **a** Expression of the 5 lncRNA conserved between human and chicken. **b** Expression of all lncRNAs (pale colors) in the different tissues against all the 5 lncRNA studied here. Embryo (E), liver (L), adipose tissue (A), hypothalamus (H). Top: expression in human with *n* = 3 (embryo not represented), bottom: expression in chicken with *n* = 16. **: *p* value< 5% ***: *p* value< 1%

We then studied the expression of these five lncRNA in three tissues known to be involved in lipid metabolism, using human [110] and chicken samples. The tissues chosen were the liver and adipose tissue as key organs/tissues for lipid synthesis and triglyceride storage. The third tissue is the hypothalamus because of its central role in energy homeostasis via the regulation of food intake and energy expenditure [11]. The chicken embryo was also studied because many lncRNAs were described as expressed during embryonic development [111–113]. The expression of the 5 lncRNAs is shown in Fig. 5a. In chicken the 5 lncRNAs were overexpressed in the 3 tissues and in the embryo compared to the total lncRNA expressions (t-test; *p*-value < 0.05), the median of their expression being equivalent to the third quartile of the total lncRNA expression (Fig. 5b). LncRNAs are known to be very poorly expressed compared to protein-coding genes (10 to 100 times less [14, 20]). This observation shows that the lncRNAs described so far are generally more expressed than the whole modelled lncRNAs. *PVT1* and *SRA1* were well expressed in all 3 tissues, both in human and chicken. *DHCR24-DT* was overexpressed in the liver of both species compared to the other tissues. On the contrary, *CRNDE* was not expressed in human, at least in the tissues and samples analyzed. Finally, *NFIA-AS1* was not expressed in any of the 3 tissues analyzed, whatever the species. This does not preclude for expression in other tissues, because Hu et al. have shown a link between this gene and lipids in macrophage cells [86].

## Discussion

To our knowledge, previous reviews taken together described 27 lncRNAs involved in lipid metabolism. An extensive research has allowed us to report 33 additional lncRNAs, the majority of which identified in human or mouse, resulting in a total of 60 long noncoding genes having a role in lipid metabolism. Such a research has to be meticulous because of the multiple aliases intra and between species and is tedious and time-consuming.

In addition to gathering these 60 genes as related to the lipid metabolism, we demonstrated experimentally that two lncRNA close and on same strand with the *SCD* and *FADS2* protein-coding genes and called by the authors as *ALDBGALG00000000505049* and *FLRL7* related to the lipid metabolism, were spurious. We also made a lncRNA nomenclature revision and propose to rename some genes according to the official rules of the HUGO gene nomenclature committee [32].

We then performed a comparative analysis to identify orthologous genes between phylogenetically distant species. Such a approach allows to make use of the knowledge obtained in one species to infer the presence on lncRNAs in other species not examined so far. In particular, it facilitates the understanding of the biological role by working on species in which gene manipulations are easier. Unfortunately, this research is difficult for lncRNAs because these genes are very poorly conserved in sequence between relatively distant species; such research required synteny approaches to study the conservation of the local genomic structure around the gene of interest. We identified, out of the 60 lncRNAs, 5 lncRNAs conserved within amniotes (320Myr), including two conserved within vertebrates (440Myr). Such results are consistent with other studies; some articles reported less than 20% of lncRNAs conserved across mammals [114, 115]. Interestingly, we showed that lncRNAs described so far are more expressed compared to the whole lncRNAs present in the gene databases; the reason is likely technical since it is easier to work on lncRNAs that are more expressed.

## Conclusions

Such a catalogue of 60 genes is interesting, first, by providing new regulators of a complex lipid metabolism known to be highly regulated at the mRNA levels [42, 76]. Second, because the lipid metabolism is particularly important in human health through the numerous diseases related to this metabolism (obesity, cardiovascular diseases, hepatic steatosis ...). Third, because this metabolism is also important in livestock species where it is linked to economically important traits, such as intramuscular fat content, meat quality traits [116–118] and ectopic fat deposition and economic carcass values. It should be noted that more than ^2^/_3_ of the 60 lncRNAs reported in this review were discovered in the last 2 years and we have no doubt that this first set will be rapidly enriched in the coming years.

## Methods

### Literature review

The state of the art regarding lncRNA potentially involved in lipid metabolism was obtained by expert curation of the literature database Pubmed. In this way, lipid metabolism was defined as the including synthesis, degradation and transport of different types of lipids, with a particular interest for fatty acids and cholesterol. The keyword literature search was therefore conducted by combining the terms “lncRNA” and one of the following terms associated to apolipoprotein or to the 8 lipid classes: sterol (cholesterol), prenol, fatty acid, acylglycerol (triglyceride), phosphoglyceride, glycolipids, polyketides and sphingolipids.

### Syntenic conservation between the chicken and the species where the lncRNA was discovered

The syntenic conservation analysis was performed for all 60 lncRNA genes identified in the first step, using the following annotation reference databases: Ensembl v.92, NCBI release 109, NONCODEv5 [18], ALDB v1.0 [119], completed by the new lncRNA models that we have previously published for chicken [20, 28]. Long noncoding gene conservation by synteny analysis between human/mouse and chicken was performed according to three criteria used in the following order of priority: 1. the lncRNA in human/mouse genome is surrounded by two neighboring protein-coding genes for which a 1-to-1 orthologous gene is available in the chicken genome. We considered the lncRNA as syntenically conserved if this lncRNA was also found in chicken between these two orthologous protein-coding genes, in the same orientation and order and same relative position and/or intron-exon structure. 2. The lncRNA in human/mouse genome is a close divergent lncRNA (< 1 kb) or antisense from a protein-coding gene with a 1-to-1 orthologous gene in the chicken genome; 3. the lncRNA in human/mouse genome is hosting small noncoding genes. Such a lncRNA was syntenically conserved if a lncRNA in chicken is hosting small noncoding genes with the same numbered-names.

### Syntenic conservation between eight species

The syntenic conservation analysis was further performed across eight species (human, mouse, goat, cattle, pig, chicken, xenope and zebrafish) for the subset of 5 lncRNA genes, which were found conserved between the chicken and the human/mouse. We used the same three criteria aforementioned, taking the lncRNA reference databases in the following order: Ensembl v.92, NCBI annotation release 109, and the new lncRNA catalogue that we have previously published for livestock species (goat, cattle, pig) [28]. The phylogenetic tree was performed with the phyloT v2018.3 tool (https://phylot.biobyte.de/), which generates phylogenetic trees based on NCBI species names and taxonomy. The tree visualization was carried out with the iTOL v4.2.3 tool [120].

### Tissue expression analysis in chicken and human

The human expression raw data from liver, white adipose tissue and brain were obtained from RNAseq data (2 × 100 bp) from three subjects (approximatively 18 M read-pairs per sample) chosen from the NCBI BioProject accession number PRJEB4337 [110]. The aim of Fagerberg et al. was to study tissue-specific expression of genes from 27 healthy human tissues. The chicken liver and abdominal adipose tissue expression data were obtained from RNAseq data (2 × 100 bp) from 16 animals (about 55 M read-pairs per sample for liver and 70 M for adipose tissue) extracted from our PRJNA330615 project [20]. The aim of Muret et al. was to model new lncRNA in chicken in these two tissues. For the hypothalamus and the embryo in chicken, the raw data used correspond to RNAseq data (2 × 150 bp) from 16 individuals (approximately 50 M read-pairs per sample - PRJEB28745). For these 2 species and all the tissues mentioned, normalized RPKM data were used.

### RT-PCR for remodeling gene structure

Total RNAs was extracted from the liver of 48 chickens (from 9-weeks broilers) and mice (C57BL/6) as described by Desert et al. [4] and reverse-transcribed using High-Capacity cDNA Reverse Transcription kit (Applied Biosystems, Foster City, CA) following manufacturer’s instructions. cDNAs was treated with DNAseI (kit Ambion – RNAse-free) and diluted 1:10 for specific PCR amplification using the primers defined in Additional file 2. The amplification specificity was confirmed by sequencing. The amplification fragment lengths were verified on 2% agarose gel using the marker SmartLadder (Eurogentec MW1700_10).

## Supplementary information

**Additional file 1:** Gene structure of HMGCR. In blue: HMGCR isoforms from Ensembl. In green: lncRNA AT102202 with 3 exons in common with HMGCR isoforms. In purple: position of the siRNA used by Liu et al. for the AT10202 knockdown and in red, primers used for the expression evaluation. Empty blocks represent noncoding regions. HMGCR isoforms that encode the HMGCR protein are the first 3 models.

**Additional file 2:** List of primers used for model validation by RT-PCR.

## Data Availability

RNA-Seq data for chicken livers and adipose tissues are available on SRA at project PRJNA330615 and for chicken hypothalamus and embryos on ENA at project PRJEB28745.

## Abbreviations

5-8F: Human nasopharyngeal carcinoma cell line
ABCA1/ABCG5: ATP-Binding Cassette A1/G5
ACACA/ACACB: Acetyl-CoA Carboxylase Alpha/Bêta
ACADS/ACADVL: Acyl-CoA Dehydrogenase Short chain/Very Long chain
ACAT2: Acetyl-CoA AcetylTransferase 2
ACC1: Acetyl-CoA Carboxylase 1 (ACC1 – ACACA)
ACLY: ATP Citrate LYase
ACOT9: Acyl-CoA Thioesterase 9
ACOX1: Acyl-CoA OXidase 1
ACSL1: Acyl-CoA Synthetase Long chain 1
Akt: PKB (Protein Kinase B)
ANKHD1: Ankyrin repeat and KH Domain-containing protein 1
ANRIL: Antisense Non-coding RNA in the INK4 Locus
APO genes: APOlipoprotein genes
AS: AntiSense
ASC: Adipose Stromal Cells
ATGL: Adipose triglyceride lipase
B4GALT1: Beta-1,4-GALactosylTransferase 1
BCL1/BCL2: B-Cell Lymphoma protein 1/2
Blnc1: Brown fat LNCRNA 1
CASIMO1: Cancer Associated Small Integral Membrane ORF 1
CBRH-7919: Rat hepatocarcinoma cell line
CD36: Cluster of Differentiation 36
cDNA: complementary DNA
CDS1: CDP-Diacylglycerol Synthase 1
CEBPA: CCAAT/Enhancer-Binding Protein Alpha
ceRNA: competing endogenous RNA
ChIP: Chromatin ImmunoPrecipitation
ChIRP: Chromatin Isolation by RNA Purification
CNE2: Human nasopharyngeal carcinoma cell line
CPT1A: Carnitine PalmitoylTransferase 1A
CRNDE: ColoRectal Neoplasia Differentially Expressed
CRP: C-Reactive Protein
CYP51A1/7A1/8B1: Cytochrome P450 51A1/7A1/8B1
DAG: DiAcylGlycerol
DGAT2: DiGlyceride AcylTransferase 2
DGKA: DiacylGlycerol Kinase Alpha
DHCR24: 24-DeHydroCholesterol Reductase
DNA: DesoxyriboNucleic Acid
DT: Divergent transcript
EDF1: Endothelial Differentiation related Factor 1
ELOVL6: ELongation Of Very Long chain fatty acids protein 6
FA: Fatty Acid
FABP4/5: Fatty Acid-Binding Protein 4/5
FADS2: Fatty Acid DeSaturase 2
FASN: Fatty Acid Synthase
FDPS: Farnesyl DiPhosphate Synthase
FLRL3/5/8: Fatty Liver Related LncRNA 3/5/8
FXR: Farnesoid X Receptor; from NR1H4 gene
GENE-AS#: Antisense of GENE
GENE-DT: Divergent Transcript from GENE
GPAT3: Glycerol-3-Phosphate AcylTransferase 3
GPR119: G Protein-coupled Receptor 119
GWAS: Genome-Wide Association Study
H3K27: Histone 3, Lysine 27
H3K27me3: trimethylation of H3K27
HAGLR: HOXD Antisense Growth-associated Long non-coding RNA
HCC: HepatoCellular Carcinoma
HCT116, HCT 119, HT29: Human colorectal carcinoma cell line
HDL: High-Density
HEK-293 T: Human embryonic kidney cell line
HeLa229: Human cervix adenocarcinoma cell line
HEM-1: Human melanoma cell line
HepG2: Human hepatocarcinoma cell line
HMGCR: 3-Hydroxy-3-Methylglutaryl-CoA Reductase
HMGCS: 3-Hydroxy-3-Methylglutaryl-CoA Synthase
hnRNP: Heterogeneous Nuclear RiboNucleoProtein
HOTAIR: HOX Transcript Antisense RNA
HOXC6: Homeobox protein C6
HSD17B7: 17-beta-HydroxySteroid Dehydrogenase
HUGO: HUman Genome Organisation
Huh7: Human hepatocarcinoma cell line
HULC: Highly Upregulated in Liver Cancer
HUVEC: Human Umbilical Vein Endothelial Cells
IDI1: Isopentenyl-diphosphate Delta Isomerase 1
IGF2BP2: Insulin-like Growth Factor 2 Binding Protein 2
IL-1β/6: Interleukin 1β/6
INPP5D: INositol Polyphoshate-5-Phosphatase D
IRX5: Iroquois Homeobox 5
KD: Knockdown
KO: Knockout
LDL: Low-Density Lipoprotein
LEP: Leptin
LeXis: Liver-Expressed LXR-Induced Sequence
linc-ADAL: lincRNA for ADipogenesis And Lipogenesis
LINCADL: LincRNA for ADipogenesis And Lipogenesis
lincRNA-DYNLRB2–2: linRNA-DYNein Light chain RoadBlock-type 2, transcript 2
LINK-A: Long Intergenic Noncoding RNA for Kinase Activation
LISPR1: Long Intergenic noncoding RNA antisense to S1PR1
LIVAR: Liver cell Viability Associated lncRNA
lncARSR: lncRNA Activated in RCC with Sunitinib Resistance
lncHR1: lncRNA HCV Regulated 1
lnc-KDM5D-4: lncRNA-Lysine DeMethylase 5D, transcript 4
lncLSTR: lncRNA Liver-Specific Triglyceride Regulator
lncRNA: long noncoding RNA
lncSHGL: lncRNA Suppressor of Hepatic Gluconeogenesis and Lipogenesis
LNMICC: lncRNA associated with Lymph Node Metastasis In Cervical Cancer
LPCAT3: LysoPhosphatidylCholine AcylTransferase 3
LPIN1/2: Lipin 1/2
LPL: LipoProtein Lipase
LSD1: Lysine (K)-Specific Demethylase 1; from KDM1A gene
LSS: Lanosterol Synthase
LXR: Liver X Receptor; from NR1H2/3 genes
MALAT1: Metastasis Associated in Lung Adenocarcinoma Transcript 1
MCF-7: Human breast cancer cell line
MEG3: Maternally Expressed Gene 3
MeXis: Macrophage-Expressed LXR-Induced Sequence
MG-63: Human osteosarcoma cell line
MGI: Mouse Genome Informatics
miRNA: micro RNA
MVD: MeValonate diphosphate Decarboxylase
MVK: MeValonate Kinase
NAFLD: Non-Alcoholic Fatty Liver Disease
NASH: Non-Alcoholic Steato-Hepatitis
NEAT1: Nuclear Enriched Abundant Transcript 1
NF1: NeuroFibromin 1
NFIA: Nuclear Factor I A
NRF2: NFE2-Related Factor 2; from NFE2L2 gene
NRON: Noncoding RNA Repressor Of NFAT
NSCLC: Non-Small Cell Lung Cancer
OLMALINC: OLigodendrocyte Maturation-Associated Long Intergenic NonCoding RNA
ORF: Open Reading Frame
Ov.: Overexpression
Ox-LDL: Oxidized LDL
PCR: Polymerase Chain Reaction
PCSK9: Proprotein Convertase Subtilisin/Kexin Type 9
PI4KB: Phosphatidylinositol 4-Kinase Beta
PIP3: Phosphatidylinositol (3,4,5)-trisphosphate
PL: Phospholipid
PLA2G1B: PhosphoLipase A2, Group 1B
PLCB3/G1: PhosphoLipase C Beta 3/Gamma 1
PMVK: PhosphoMeValonate Kinase
PNPLA2: PatatiN-like PhosphoLipase A2
PPARA/G: Peroxisome Proliferator-Activated Receptors Alpha/Gamma
PRC2: Polycomb Repressive Complex 2
PTBP1: Polypyrimidine Tract Binding Protein 1
PVT1: Plasmacytoma Variant Translocation 1
qPCR: quantitative PCR
RALY: hnRNP Associated with Lethal Yellow protein
RIP: RNA ImmunoPrecipitation
RNA: RiboNucleic Acid
RNCR3: Retinal Non-Coding RNA 3
RPKM: Reads Per Kilobase of transcript, per Million mapped reads
RT-PCR: Reverse Transcription PCR
S1PR1: Sphingosine-1-Phosphate Receptor 1
SAF: Antisense of FAS (Fas Cell Surface Death Receptor)
SCD: Stearoyl-CoA Desaturase
SHP: Small Heterodimer Partner
siRNA: small interfering RNA
SMCR2: Smith-Magenis syndrome Chromosome Region, candidate 2
SMIM22: SMall Integral Membrane protein 22
SNHG16: Small Noncoding Host Gene 16
SNP: Single Nucleotide Polymorphism
SQLE: SQuaLene Epoxidase
SRA1: Steroid Receptor RNA Activator 1
SRAP: Steroid Receptor RNA Activator Protein
SREBF1/2: Sterol Regulatory Element Binding transcription Factor 1/2
SUZ12: SUppressor of Zeste 12
TAG: TriAcylGlycerol
TG: TriGlycerides
THP-1: Human leukemic monocyte cell line
TNBC: Triple-Negative Breast Cancer
TNFa: Tumor Necrosis Factor
TRIB1/3: TRIBbles homolog 1/3
TRIBAL: TRIB1-Associated Locus
U2OS: Human osteosarcoma cell line
uc.372: ultra Conserved 372
VLDL: Very Low-Density

## References

[1] Bergen WG, Mersmann HJ (2005). Comparative aspects of lipid metabolism: impact on contemporary research and use of animal models. J Nutr.

[2] Parrish CC (2013). Lipids in marine ecosystems. ISRN Ocenaogr.

[3] Li N, Xu C, Li-Beisson Y, Philippar K (2016). Fatty acid and lipid transport in plant cells. Trends Plant Sci.

[4] Desert C, Baéza E, Aite M, Boutin M, Le Cam A, Montfort J (2018). Multi-tissue transcriptomic study reveals the main role of liver in the chicken adaptive response to a switch in dietary energy source through the transcriptional regulation of lipogenesis. BMC Genomics.

[5] Sato K, Kamada T (2011). Regulation of bile acid, cholesterol, and fatty acid synthesis in chicken primary hepatocytes by different concentrations of T0901317, an agonist of liver X receptors. Comp Biochem Physiol A Mol Integr Physiol.

[6] Hillgartner FB, Salati LM, Goodridge AG (1995). Physiological and molecular mechanisms involved in nutritional regulation of fatty acid synthesis. Physiol Rev.

[7] Greene DH, Selivonchick DP (1987). Lipid metabolism in fish. Prog Lipid Res.

[8] Ye J, DeBose-Boyd RA (2011). Regulation of cholesterol and fatty acid synthesis. Cold Spring Harb Perspect Biol.

[9] Viturro E, Koenning M, Kroemer A, Schlamberger G, Wiedemann S, Kaske M (2009). Cholesterol synthesis in the lactating cow: induced expression of candidate genes. J Steroid Biochem Mol Biol.

[10] Shreni KD, Jafri AK (1977). Seasonal variations in the total cholesterol content of the liver of cat fish Heteropnuestes fossilis (Bloch). Fish Technol.

[11] Diéguez C, Vazquez MJ, Romero A, López M, Nogueiras R (2011). Hypothalamic control of lipid metabolism: focus on leptin, ghrelin and melanocortins. Neuroendocrinology.

[12] Harrow J, Frankish A, Gonzalez JM, Tapanari E, Diekhans M, Kokocinski F (2012). GENCODE: the reference human genome annotation for the ENCODE project. Genome Res.

[13] Mudge JM, Harrow J (2015). Creating reference gene annotation for the mouse C57BL6/J genome assembly. Mamm Genome.

[14] Derrien T, Johnson R, Bussotti G, Tanzer A, Djebali S, Tilgner H (2012). The GENCODE v7 catalog of human long noncoding RNAs: analysis of their gene structure, evolution, and expression. Genome Res.

[15] Morris KV, Mattick JS (2014). The rise of regulatory RNA. Nat Rev Genet.

[16] Mercer TR, Dinger ME, Mattick JS (2009). Long non-coding RNAs: insights into functions. Nat Rev Genet.

[17] van Solingen C, Scacalossi KR, Moore KJ (2018). Long noncoding RNAs in lipid metabolism. Curr Opin Lipidol.

[18] Fang S, Zhang L, Guo J, Niu Y, Wu Y, Li H (2018). NONCODEV5: a comprehensive annotation database for long non-coding RNAs. Nucleic Acids Res.

[19] Volders P-J, Verheggen K, Menschaert G, Vandepoele K, Martens L, Vandesompele J (2015). An update on LNCipedia: a database for annotated human lncRNA sequences. Nucleic Acids Res.

[20] Muret K, Klopp C, Wucher V, Esquerré D, Legeai F, Lecerf F, et al. Long noncoding RNA repertoire in chicken liver and adipose tissue. Genet Sel Evol. 2017;49. 10.1186/s12711-016-0275-0. 10.1186/s12711-016-0275-0 PMC5225574 28073357

[21] Gloss BS, Dinger ME (2016). The specificity of long noncoding RNA expression. Biochim Biophys Acta.

[22] Kang Y-J, Yang D-C, Kong L, Hou M, Meng Y-Q, Wei L (2017). CPC2: a fast and accurate coding potential calculator based on sequence intrinsic features. Nucleic Acids Res.

[23] Kong L, Zhang Y, Ye Z-Q, Liu X-Q, Zhao S-Q, Wei L (2007). CPC: assess the protein-coding potential of transcripts using sequence features and support vector machine. Nucleic Acids Res.

[24] Wang L, Park HJ, Dasari S, Wang S, Kocher J-P, Li W (2013). CPAT: coding-potential assessment tool using an alignment-free logistic regression model. Nucleic Acids Res.

[25] Lin MF, Jungreis I, Kellis M (2011). PhyloCSF: a comparative genomics method to distinguish protein coding and non-coding regions. Bioinformatics.

[26] Wucher V, Legeai F, Hedan B, Rizk G, Lagoutte L, Leeb T (2016). FEELnc: a tool for Long non-coding RNAs annotation and its application to the dog transcriptome.

[27] Hezroni H, Koppstein D, Schwartz MG, Avrutin A, Bartel DP, Ulitsky I (2015). Principles of long noncoding RNA evolution derived from direct comparison of transcriptomes in 17 species. Cell Rep.

[28] Foissac S, Djebali S, Munyard K, Villa-Vialaneix N, Rau A, Muret K, et al. Livestock genome annotation: transcriptome and chromatin structure profiling in cattle, goat, chicken and pig. bioRxiv. 2018. 10.1101/316091.

[29] Ulitsky I (2016). Evolution to the rescue: using comparative genomics to understand long non-coding RNAs. Nat Rev Genet.

[30] Ulitsky I, Shkumatava A, Jan CH, Sive H, Bartel DP (2011). Conserved function of lincRNAs in vertebrate embryonic development despite rapid sequence evolution. Cell.

[31] Chen Z (2016). Progress and prospects of long noncoding RNAs in lipid homeostasis. Mol Metab.

[32] Wright MW (2014). A short guide to long non-coding RNA gene nomenclature. Hum Genomics.

[33] Zhou T, Ding J, Wang X-A, Zheng X (2016). Long noncoding RNAs and atherosclerosis. Atherosclerosis.

[34] Smekalova EM, Kotelevtsev YV, Leboeuf D, Shcherbinina EY, Fefilova AS, Zatsepin TS (2016). lncRNA in the liver: prospects for fundamental research and therapy by RNA interference. Biochimie.

[35] Ananthanarayanan M (2016). A novel long noncoding RNA regulating cholesterol and bile acid homeostasis: a new kid on the block and a potential therapeutic target?. Hepatology.

[36] Zhao Y, Wu J, Liangpunsakul S, Wang L (2017). Long non-coding RNA in liver metabolism and disease: current status. Liver Res.

[37] Sallam T, Jones M, Thomas BJ, Wu X, Gilliland T, Qian K (2018). Transcriptional regulation of macrophage cholesterol efflux and atherogenesis by a long noncoding RNA. Nat Med.

[38] Zeng Y, Ren K, Zhu X, Zheng Z, Yi G (2018). Long noncoding RNAs: advances in lipid metabolism. Adv Clin Chem.

[39] Liu G, Zheng X, Xu Y, Lu J, Chen J, Huang X (2015). Long non-coding RNAs expression profile in HepG2 cells reveals the potential role of long non-coding RNAs in the cholesterol metabolism. Chin Med J.

[40] Lillycrop K, Murray R, Cheong C, Teh AL, Clarke-Harris R, Barton S (2017). ANRIL promoter DNA methylation: a perinatal marker for later adiposity. EBioMedicine.

[41] Halley P, Kadakkuzha BM, Faghihi MA, Magistri M, Zeier Z, Khorkova O (2014). Regulation of the apolipoprotein gene cluster by a long noncoding RNA. Cell Rep.

[42] Qin W, Li X, Xie L, Li S, Liu J, Jia L (2016). A long non-coding RNA, APOA4-AS, regulates APOA4 expression depending on HuR in mice. Nucleic Acids Res.

[43] Chen Y, Huang H, Xu C, Yu C, Li Y (2017). Long non-coding RNA profiling in a non-alcoholic fatty liver disease rodent model: new insight into pathogenesis. Int J Mol Sci.

[44] Zhao X-Y, Li S, DelProposto JL, Liu T, Mi L, Porsche C (2018). The long noncoding RNA Blnc1 orchestrates homeostatic adipose tissue remodeling to preserve metabolic health. Mol Metab.

[45] Polycarpou-Schwarz M, Groß M, Mestdagh P, Schott J, Grund SE, Hildenbrand C (2018). The cancer-associated microprotein CASIMO1 controls cell proliferation and interacts with squalene epoxidase modulating lipid droplet formation. Oncogene.

[46] Ellis BC, Graham LD, Molloy PL (2014). CRNDE, a long non-coding RNA responsive to insulin/IGF signaling, regulates genes involved in central metabolism. Biochim Biophys Acta.

[47] Cao C, Fan R, Zhao J, Zhao X, Yang J, Zhang Z (2017). Impact of exudative diathesis induced by selenium deficiency on LncRNAs and their roles in the oxidative reduction process in broiler chick veins. Oncotarget.

[48] Reddy MA, Chen Z, Park JT, Wang M, Lanting L, Zhang Q (2014). Regulation of inflammatory phenotype in macrophages by a diabetes-induced long noncoding RNA. Diabetes.

[49] Yang L, Li P, Yang W, Ruan X, Kiesewetter K, Zhu J (2016). Integrative transcriptome analyses of metabolic responses in mice define pivotal LncRNA metabolic regulators. Cell Metab.

[50] Liu C, Yang Z, Wu J, Zhang L, Lee S, Shin D-J (2018). Long noncoding RNA H19 interacts with polypyrimidine tract-binding protein 1 to reprogram hepatic lipid homeostasis. Hepatology.

[51] Lu C, Ma J, Cai D (2017). Increased HAGLR expression promotes non-small cell lung cancer proliferation and invasion via enhanced de novo lipogenesis. Tumour Biol.

[52] Ma D-D, Yuan L-L, Lin L-Q (2017). LncRNA HOTAIR contributes to the tumorigenesis of nasopharyngeal carcinoma via up-regulating FASN. Eur Rev Med Pharmacol Sci.

[53] Huang C, Hu Y-W, Zhao J-J, Ma X, Zhang Y, Guo F-X (2016). Long noncoding RNA HOXC-AS1 suppresses ox-LDL-induced cholesterol accumulation through promoting HOXC6 expression in THP-1 macrophages. DNA Cell Biol.

[54] Cui M, Xiao Z, Wang Y, Zheng M, Song T, Cai X (2015). Long noncoding RNA HULC modulates abnormal lipid metabolism in hepatoma cells through an miR-9-mediated RXRA signaling pathway. Cancer Res.

[55] Sallam T, Jones MC, Gilliland T, Zhang L, Wu X, Eskin A (2016). Feedback modulation of cholesterol metabolism by the lipid-responsive non-coding RNA LeXis. Nature.

[56] Zhang X, Wu J, Wu C, Chen W, Lin R, Zhou Y (2018). The LINC01138 interacts with PRMT5 to promote SREBP1-mediated lipid desaturation and cell growth in clear cell renal cell carcinoma. Biochem Biophys Res Commun.

[57] Zhang X, Xue C, Lin J, Ferguson JF, Weiner A, Liu W (2018). Interrogation of nonconserved human adipose lincRNAs identifies a regulatory role of linc-ADAL in adipocyte metabolism. Sci Transl Med.

[58] Hu Y-W, Yang J-Y, Ma X, Chen Z-P, Hu Y-R, Zhao J-Y (2014). A lincRNA-DYNLRB2-2/GPR119/GLP-1R/ABCA1-dependent signal transduction pathway is essential for the regulation of cholesterol homeostasis. J Lipid Res.

[59] Lin A, Hu Q, Li C, Xing Z, Ma G, Wang C (2017). The LINK-A lncRNA interacts with PtdIns(3,4,5)P3 to hyperactivate AKT and confer resistance to AKT inhibitors. Nat Cell Biol.

[60] Josipovic I, Pflüger B, Fork C, Vasconez AE, Oo JA, Hitzel J (2018). Long noncoding RNA LISPR1 is required for S1P signaling and endothelial cell function. J Mol Cell Cardiol.

[61] Atanasovska B, Rensen SS, van der Sijde MR, Marsman G, Kumar V, Jonkers I (2017). A liver-specific long noncoding RNA with a role in cell viability is elevated in human nonalcoholic steatohepatitis. Hepatology.

[62] Tristán-Flores FE, Guzmán P, Ortega-Kermedy MS, Cruz-Torres G, de la Rocha C, Silva-Martínez GA (2018). Liver X receptor-binding DNA motif associated with atherosclerosis-specific DNA methylation profiles of Alu elements and neighboring CpG Islands. J Am Heart Assoc.

[63] Zhang M, Chi X, Qu N, Wang C (2018). Long noncoding RNA lncARSR promotes hepatic lipogenesis via Akt/SREBP-1c pathway and contributes to the pathogenesis of nonalcoholic steatohepatitis. Biochem Biophys Res Commun.

[64] Huang J, Chen S, Cai D, Bian D, Wang F (2018). Long noncoding RNA lncARSR promotes hepatic cholesterol biosynthesis via modulating Akt/SREBP-2/HMGCR pathway. Life Sci.

[65] Lan X, Yan J, Ren J, Zhong B, Li J, Li Y (2016). A novel long noncoding RNA Lnc-HC binds hnRNPA2B1 to regulate expressions of Cyp7a1 and Abca1 in hepatocytic cholesterol metabolism. Hepatology.

[66] Li D, Cheng M, Niu Y, Chi X, Liu X, Fan J (2017). Identification of a novel human long non-coding RNA that regulates hepatic lipid metabolism by inhibiting SREBP-1c. Int J Biol Sci.

[67] Molina E, Chew GS, Myers SA, Clarence EM, Eales JM, Tomaszewski M (2017). A novel Y-specific long non-coding RNA associated with cellular lipid accumulation in HepG2 cells and atherosclerosis-related genes. Sci Rep.

[68] Lo KA, Huang S, Walet ACE, Zhang Z-C, Leow MK-S, Liu M (2018). Adipocyte long-noncoding RNA transcriptome analysis of obese mice identified Lnc-leptin, which regulates leptin. Diabetes.

[69] Li P, Ruan X, Yang L, Kiesewetter K, Zhao Y, Luo H (2015). A liver-enriched long non-coding RNA, lncLSTR, regulates systemic lipid metabolism in mice. Cell Metab.

[70] Li H, Gu Z, Yang L, Tian Y, Kang X, Liu X (2018). Transcriptome profile analysis reveals an estrogen induced LncRNA associated with lipid metabolism and carcass traits in chickens (Gallus Gallus). Cell Physiol Biochem.

[71] Wang J, Yang W, Chen Z, Chen J, Meng Y, Feng B (2018). Long noncoding RNA lncSHGL recruits hnRNPA1 to suppress hepatic gluconeogenesis and lipogenesis. Diabetes.

[72] Shang C, Wang W, Liao Y, Chen Y, Liu T, Du Q (2018). LNMICC promotes nodal metastasis of cervical Cancer by reprogramming fatty acid metabolism. Cancer Res.

[73] Lu M-C, Yu H-C, Yu C-L, Huang H-B, Koo M, Tung C-H (2016). Increased expression of long noncoding RNAs LOC100652951 and LOC100506036 in T cells from patients with rheumatoid arthritis facilitates the inflammatory responses. Immunol Res.

[74] Ghanbari M, Peters MJ, de Vries PS, Boer CG, van Rooij JGJ, Lee Y-C (2018). A systematic analysis highlights multiple long non-coding RNAs associated with cardiometabolic disorders. J Hum Genet.

[75] Yan C, Chen J, Chen N (2016). Long noncoding RNA MALAT1 promotes hepatic steatosis and insulin resistance by increasing nuclear SREBP-1c protein stability. Sci Rep.

[76] Zhang L, Yang Z, Trottier J, Barbier O, Wang L (2017). Long noncoding RNA MEG3 induces cholestatic liver injury by interaction with PTBP1 to facilitate shp mRNA decay. Hepatology.

[77] Zhu X, Wu Y-B, Zhou J, Kang D-M (2016). Upregulation of lncRNA MEG3 promotes hepatic insulin resistance via increasing FoxO1 expression. Biochem Biophys Res Commun.

[78] Wang X, Wang J (2018). High-content hydrogen water-induced downregulation of miR-136 alleviates non-alcoholic fatty liver disease by regulating Nrf2 via targeting MEG3. Biol Chem.

[79] Huang-Fu N, Cheng J-S, Wang Y, Li Z-W, Wang S-H (2018). Neat1 regulates oxidized low-density lipoprotein-induced inflammation and lipid uptake in macrophages via paraspeckle formation. Mol Med Rep.

[80] Liu X, Liang Y, Song R, Yang G, Han J, Lan Y (2018). Long non-coding RNA NEAT1-modulated abnormal lipolysis via ATGL drives hepatocellular carcinoma proliferation. Mol Cancer.

[81] Liu Y, Ji Y, Li M, Wang M, Yi X, Yin C (2018). Integrated analysis of long noncoding RNA and mRNA expression profile in children with obesity by microarray analysis. Sci Rep.

[82] Benhammou JN, Ko A, Alvarez M, Kaminska D, Pihlajamäki J, Pisegna JR (2018). Su1496 - The Long Intergenic, Non-Coding RNA, Olmalinc, Influences Lipid Metabolism in Hepatocytes by Regulating SREBP1 and SREBP2, and may Play a Critical Role in the Development of NAFLD. Gastroenterology.

[83] Zhou Q, Chen F, Zhao J, Li B, Liang Y, Pan W (2016). Long non-coding RNA PVT1 promotes osteosarcoma development by acting as a molecular sponge to regulate miR-195. Oncotarget.

[84] Shan K, Jiang Q, Wang X-Q, Wang Y-N-Z, Yang H, Yao M-D (2016). Role of long non-coding RNA-RNCR3 in atherosclerosis-related vascular dysfunction. Cell Death Dis.

[85] Mitchel K, Theusch E, Cubitt C, Dosé AC, Stevens K, Naidoo D (2016). RP1-13D10.2 is a novel modulator of statin-induced changes in cholesterol. Circ Cardiovasc Genet.

[86] Hu Y-W, Zhao J-Y, Li S-F, Huang J-L, Qiu Y-R, Ma X (2015). RP5-833A20.1/miR-382-5p/NFIA-dependent signal transduction pathway contributes to the regulation of cholesterol homeostasis and inflammatory reaction. Arterioscler Thromb Vasc Biol.

[87] Qi X, Shao M, Sun H, Shen Y, Meng D, Huo W (2017). Long non-coding RNA SNHG14 promotes microglia activation by regulating miR-145-5p/PLA2G4A in cerebral infarction. Neuroscience.

[88] Christensen LL, True K, Hamilton MP, Nielsen MM, Damas ND, Damgaard CK (2016). SNHG16 is regulated by the Wnt pathway in colorectal cancer and affects genes involved in lipid metabolism. Mol Oncol.

[89] Mazar J, Zhao W, Khalil AM, Lee B, Shelley J, Govindarajan SS (2014). The functional characterization of long noncoding RNA SPRY4-IT1 in human melanoma cells. Oncotarget.

[90] Chen G, Yu D, Nian X, Liu J, Koenig RJ, Xu B (2016). LncRNA SRA promotes hepatic steatosis through repressing the expression of adipose triglyceride lipase (ATGL). Sci Rep.

[91] Liu S, Sheng L, Miao H, Saunders TL, MacDougald OA, Koenig RJ (2014). SRA gene knockout protects against diet-induced obesity and improves glucose tolerance. J Biol Chem.

[92] Douvris A, Soubeyrand S, Naing T, Martinuk A, Nikpay M, Williams A (2014). Functional analysis of the TRIB1 associated locus linked to plasma triglycerides and coronary artery disease. J Am Heart Assoc.

[93] Guo J, Fang W, Sun L, Lu Y, Dou L, Huang X (2018). Ultraconserved element uc.372 drives hepatic lipid accumulation by suppressing miR-195/miR4668 maturation. Nat Commun.

[94] Huang W, Zhang X, Li A, Xie L, Miao X (2017). Differential regulation of mRNAs and lncRNAs related to lipid metabolism in two pig breeds. Oncotarget.

[95] Zhao X-Y, Xiong X, Liu T, Mi L, Peng X, Rui C (2018). Long noncoding RNA licensing of obesity-linked hepatic lipogenesis and NAFLD pathogenesis. Nat Commun.

[96] Ruan X, Li P, Cangelosi A, Yang L, Cao H (2016). A long non-coding RNA, lncLGR, regulates hepatic Glucokinase expression and glycogen storage during fasting. Cell Rep.

[97] Willingham AT, Orth AP, Batalov S, Peters EC, Wen BG, Aza-Blanc P (2005). A strategy for probing the function of noncoding RNAs finds a repressor of NFAT. Science.

[98] Bierhoff H (2018). Analysis of lncRNA-protein interactions by RNA-protein pull-down assays and RNA immunoprecipitation (RIP). Methods Mol Biol.

[99] Boivin V, Deschamps-Francoeur G, Scott MS (2018). Protein coding genes as hosts for noncoding RNA expression. Semin Cell Dev Biol.

[100] Shenker S, Miura P, Sanfilippo P, Lai EC (2015). IsoSCM: improved and alternative 3′ UTR annotation using multiple change-point inference. RNA.

[101] Fan R, Cao C, Zhao X, Shi Q, Zhao J, Xu S (2017). Downregulated long noncoding RNA ALDBGALG0000005049 induces inflammation in chicken muscle suffered from selenium deficiency by regulating stearoyl-CoA desaturase. Oncotarget.

[102] Szafron LM, Balcerak A, Grzybowska EA, Pienkowska-Grela B, Felisiak-Golabek A, Podgorska A (2015). The novel gene CRNDE encodes a nuclear peptide (CRNDEP) which is overexpressed in highly proliferating tissues. PLoS One.

[103] Lanz RB, McKenna NJ, Onate SA, Albrecht U, Wong J, Tsai SY (1999). A steroid receptor coactivator, SRA, functions as an RNA and is present in an SRC-1 complex. Cell.

[104] Liu C, Wu H-T, Zhu N, Shi Y-N, Liu Z, Ao B-X (2016). Steroid receptor RNA activator: biologic function and role in disease. Clin Chim Acta.

[105] Kawashima H, Takano H, Sugita S, Takahara Y, Sugimura K, Nakatani T (2003). A novel steroid receptor co-activator protein (SRAP) as an alternative form of steroid receptor RNA-activator gene: expression in prostate cancer cells and enhancement of androgen receptor activity. Biochem J.

[106] Cooper C, Vincett D, Yan Y, Hamedani MK, Myal Y, Leygue E (2011). Steroid receptor RNA activator bi-faceted genetic system: heads or tails?. Biochimie.

[107] Kumar S, Hedges SB (1998). A molecular timescale for vertebrate evolution. Nature.

[108] Hedges SB, Kumar S (2002). Genomics. Vertebrate genomes compared. Science.

[109] Kerner P, Ikmi A, Coen D, Vervoort M (2009). Evolutionary history of the Iroquois/Irx genes in metazoans. BMC Evol Biol.

[110] Fagerberg L, Hallström BM, Oksvold P, Kampf C, Djureinovic D, Odeberg J (2014). Analysis of the human tissue-specific expression by genome-wide integration of transcriptomics and antibody-based proteomics. Mol Cell Proteomics.

[111] Schmitz SU, Grote P, Herrmann BG (2016). Mechanisms of long noncoding RNA function in development and disease. Cell Mol Life Sci.

[112] Perry RB-T, Ulitsky I (2016). The functions of long noncoding RNAs in development and stem cells. Development.

[113] Ponting CP, Oliver PL, Reik W (2009). Evolution and functions of long noncoding RNAs. Cell.

[114] Breschi A, Gingeras TR, Guigó R (2017). Comparative transcriptomics in human and mouse. Nat Rev Genet.

[115] Le Béguec C, Wucher V, Lagoutte L, Cadieu E, Botherel N, Hédan B (2018). Characterisation and functional predictions of canine long non-coding RNAs. Sci Rep.

[116] Hocquette JF, Gondret F, Baéza E, Médale F, Jurie C, Pethick DW (2010). Intramuscular fat content in meat-producing animals: development, genetic and nutritional control, and identification of putative markers. Animal.

[117] Wood JD, Enser M, Fisher AV, Nute GR, Sheard PR, Richardson RI (2008). Fat deposition, fatty acid composition and meat quality: a review. Meat Sci.

[118] Listrat A, Lebret B, Louveau I, Astruc T, Bonnet M, Lefaucheur L (2016). How muscle structure and composition influence meat and flesh quality. ScientificWorldJournal.

[119] Li A, Zhang J, Zhou Z, Wang L, Liu Y, Liu Y (2015). ALDB: a domestic-animal long noncoding RNA database. PLoS One.

[120] Letunic I, Bork P (2016). Interactive tree of life (iTOL) v3: an online tool for the display and annotation of phylogenetic and other trees. Nucleic Acids Res.

