# Supplementary figures and images for: Long noncoding RNAs in lipid metabolism: literature review and conservation analysis across species
Source: BMC Genomics. 2019 Nov 21;20:882. doi: 10.1186/s12864-019-6093-3 (PMC6868825; doi:10.1186/s12864-019-6093-3)

*e!* HMGCR isoforms

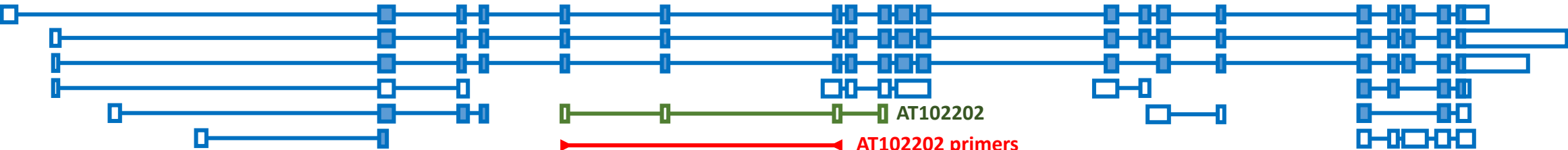

▲ siRNA

AT102202 primers

HMGCR primers

Supplement: Supplementary file 1 — Additional file 1: Gene structure of HMGCR. In blue: HMGCR isoforms from Ensembl. In green: lncRNA AT102202 with 3 exons in common with HMGCR isoforms. In purple: position of the siRNA used by Liu et al. for the AT10202 knockdown and in red, primers used for the expression evaluation. Empty blocks represent noncoding regions. HMGCR isoforms that encode the HMGCR protein are the first 3 models. [file 12864_2019_6093_MOESM1_ESM.pdf]
