# Supplementary material for: Long noncoding RNAs in lipid metabolism: literature review and conservation analysis across species
Source: BMC Genomics. 2019 Nov 21;20:882. doi: 10.1186/s12864-019-6093-3 (PMC6868825; doi:10.1186/s12864-019-6093-3)
Supplement: Supplementary file 2 — Additional file 2: List of primers used for model validation by RT-PCR. [file 12864_2019_6093_MOESM2_ESM.docx]

| Primer_name | Primers | length |
| --- | --- | --- |
| ALDBGALG-SCD models | | |
| I_ALDBGALG_ex1_forward | AGGAAGGGGTAACGTTGGAG | 20 |
| I_ALDBGALG_ex1_reverse | GGCACCAGAAGCAAGGAATC | 20 |
| II_SCD-ALDBGALG_forward | ACACGCTGGACAAAGGTTTAATG | 23 |
| II_SCD-ALDBGALG_reverse | TCATTCTCCTCACCTTCCCCT | 21 |
| III_Intergenic_forward | CCAAAGTCAGTGTGATCGTCG | 21 |
| III_Intergenic_reverse | AGTGGATAATGGTGAGGGGA | 20 |
| IV_SCD_ex5-6_forward | CCATCCTGCGCTACACCTTA | 20 |
| IV_SCD_ex5-6_reverse | AACCTTTGTCCAGCGTGTTC | 20 |
| FLRL7-FADS2 models | | |
| I_FLRL7_ex1_forward | ATGGCTGGGATTGGTACTTG | 20 |
| I_FLRL7_ex1_reverse | ATGGTGGGCATGAGAGAACT | 20 |
| II_FADS2-FLRL7_forward | TGTGGCTGGATGCTTACCTC | 20 |
| II_FADS2-FLRL7_reverse | TCCTGGACTCCTGTTCCACA | 20 |
| III_Intergenic_forward | TGTCTGCTGCCTCACATTCC | 20 |
| III_Intergenic_reverse | GGCTGACAAAGGGGGAACTC | 20 |
| IV_FADS2_ex3-4_forward | CCTTTGTCCTCGCTACCTCTCA | 22 |
| IV_FADS2_ex3-4_reverse | ATAGACAGAAAGGTGGCCATAGTCA | 25 |

**List of primers used for model validation by RT-PCR.**
